# Synergistic nanomedicine overcomes hypoxia-driven DNA repair to potentiate radiotherapy for lung adenocarcinoma

**DOI:** 10.7150/thno.132916

**Published:** 2026-03-17

**Authors:** Zhichang Liu, Yue Qiu, Jie Chen, Jue Li, Cheng Zhuang, Qiyi Feng, Jinxing Huang, Chunxiu Xiao, Xiuli Zheng, Kui Luo, Kai Xiao

**Affiliations:** 1 Laboratory of Precision Therapeutics, Department of Pulmonary and Critical Care Medicine, State Key Laboratory of Respiratory Health and Multimorbidity, Institution of Radiology and Medical Imaging, Huaxi MR Research Center (HMRRC), Department of Radiology, National Clinical Research Center for Geriatrics, State Key Laboratory of Biotherapy, Frontiers Science Center for Disease-Related Molecular Network, West China Hospital, Sichuan University, Chengdu, 610041, China.; 2 Frontier Medical Center, Tianfu Jincheng Laboratory, Chengdu, 610041, China.; 3 Psychoradiology Key Laboratory of Sichuan Province, and Research Unit of Psychoradiology, Chinese Academy of Medical Sciences, Chengdu, 610041, China.

**Keywords:** lung adenocarcinoma, radiosensitivity, DNA damage repair, hypoxia, nanomedicine, combination therapy

## Abstract

Rationale: Radiotherapy (RT) remains a mainstay for inoperable lung adenocarcinoma (LUAD), while its efficacy is frequently compromised by hypoxia-driven radioresistance. Hypoxia stabilizes hypoxia-inducible factor-1α (HIF-1α), which triggers pro-repair DNA damage response (DDR) programs. This process intensifies replication stress and ultimately enhances tumor dependence on ataxia-telangiectasia and Rad3-related (ATR)-dependent checkpoint signaling for survival. Coordinated suppression of these adaptive programs may overcome hypoxia-driven tolerance to RT and improve therapeutic responses.

Methods: The clinical relevance of HIF-1α and ATR signaling in LUAD was established through integrative bioinformatic analyses of a patient cohort. A redox-responsive polymeric nanoplatform incorporating gadolinium (Gd³⁺) and pyropheophorbide a (Ppa) was constructed to enable X-ray-activated radiodynamic therapy (RDT) and co-deliver HIF-1α siRNA with AZD6738, an ATR inhibitor. Therapeutic efficacy, radiosensitization, and mechanisms were studied *in vitro* and in a LUAD patient-derived xenograft (PDX) model.

Results: Bioinformatic analyses support the rationale for simultaneously targeting hypoxia-adaptive programs and checkpoint-mediated DDR. The nanomedicine achieves efficient co-delivery of HIF-1α siRNA and AZD6738, suppressing hypoxia-driven adaptation and impairing ATR-dependent checkpoint protection. In addition, Gd³⁺ promotes X-ray energy deposition to activate Ppa, amplifying radiodynamic reactive oxygen species (ROS) generation. These complementary biological and physicochemical actions synergistically enhance tumor cell killing and markedly improve radiosensitivity *in vitro* and *in vivo*.

Conclusions: This study establishes a synergistic nanotherapeutic strategy to concurrently disrupt the HIF-1α/ATR axis and augment radiodynamic ROS production. By integrating biological pathway inhibition with damage amplification, our strategy effectively overcomes hypoxia-mediated radioresistance, offering a promising and translatable paradigm for enhancing RT outcomes in LUAD.

## Introduction

Radiotherapy (RT) is a principal treatment modality for lung adenocarcinoma (LUAD) and it is integral to both curative and palliative management. However, its clinical efficacy is frequently compromised by intrinsic and acquired radioresistance, a multifactorial process driven predominantly by a complex tumor microenvironment (TME) 1, 2. Two mechanistically linked adaptive responses are now recognized as key drivers of radioresistance: (i) tumor hypoxia, which restricts the efficient generation of highly cytotoxic reactive oxygen species (ROS), thereby diminishing radiation-induced DNA damage; and (ii) hyperactivated DNA damage response (DDR) pathways, which facilitate the repair of radiation-induced lesions and promote tumor cell survival 3-5.

Hypoxia-inducible factor-1α (HIF-1α) is a master transcriptional regulator of the cellular response to low oxygen tension 5. Under hypoxic conditions, its stabilization drives the activation of a broad transcriptional program that supports tumor cell survival, angiogenesis, metabolic reprogramming, and invasion 6. HIF-1α overexpression is a characteristic of solid tumors and it is strongly associated with poor prognosis and resistance to RT 5, 7. Notably, RT-induced stress can stabilize HIF-1α, which in turn facilitates the repair of lethal DNA double-strand breaks (DSBs), thereby promoting survival of hypoxic tumor cells 8, 9. In addition, robust DDR signaling is activated in tumor cells to relieve genotoxic stress. Ataxia-telangiectasia and Rad3-related (ATR) kinase is a central regulator of the DDR, particularly in response to replication stress and DSBs 10, 11. Downregulation of ATR could weaken the DDR, and ATR has become an attractive target for radiosensitization 11, 12. Importantly, these pathways are interwoven: hypoxic stress can directly activate the ATR/checkpoint kinase 1 (Chk1) axis, establishing a coordinated pro-survival network that diminishes the efficacy of single-modal interventions 5, 13. Collectively, this integrated hypoxia-DDR axis constitutes a major biological barrier to effective RT for LUAD.

Strategies have been developed to overcome tumor radioresistance, such as physical dose enhancement, radiodynamic ROS amplification, and pharmacological inhibition of hypoxia- or DDR-associated pathways, while these strategies are often applied independently. These strategies could be combined within a single platform to concurrently address multiple resistance mechanisms in the same tumor cell population 14. In addition, conventional organic photosensitizers used for radiodynamic therapy (RDT) exhibit poor X-ray absorption, resulting in a suboptimal energy conversion efficiency 14. Although high-atomic-number (high-Z) nanomaterials can enhance local radiation dose deposition, they often fail to generate sufficient ROS or disrupt the molecular programs that sustain radioresistance 15, 16. Moreover, it is challenging to deliver molecular inhibitors targeting hypoxia and DDR signaling in a coordinated and co-localized manner into the same tumor cell population when they are administered separately 13, 17. Therefore, it could be therapeutically effective by simultaneously enabling efficient X-ray energy conversion into cytotoxic ROS and co-delivery of agents to suppress both hypoxia-driven adaptation and DDR-mediated survival within the same intracellular compartment.

In this context, we first established clinical and molecular rationales for the integrated approach through bioinformatic analyses. Interrogation of The Cancer Genome Atlas (TCGA) LUAD cohort reveals that elevated hypoxia-associated and DNA repair-related gene signatures are associated with poor clinical outcomes, supporting simultaneously co-targeting these adaptive programs. Transcriptomic analyses also support that irradiation could reinforce these resistance pathways. Inspired by these findings, we engineered a redox-responsive polymeric nanocarrier (PDP) as a multifunctional delivery platform to co-load small interfering RNA (siRNA) for HIF-1α and an ATR inhibitor, AZD6738, thereby achieving their concurrent intracellular delivery. To enhance radiosensitization, a gadolinium (Gd³⁺)-photosensitizer was also incorporated into the PDP platform to produce a nanomedicine, Gd@PDP-AZD-siRNA. The nanomedicine initiates a coupled sensitization cascade. Gd³⁺ enhances local energy deposition under X-ray irradiation, and the photosensitizer converts the energy into potent RDT-mediated ROS generation. By integrating ROS amplification with HIF-1α silencing and ATR inhibition, this nanomedicine directly disrupts the hypoxia-DDR resistance axis. Our study presents a multimodal therapeutic strategy to address key challenges of current radiosensitization approaches and holds considerable promise for improving RT outcomes in LUAD.

## Results

### Hypoxia-driven radioresistance in LUAD and the rationale for dual HIF-1α/ATR blockade

**Figure 1:**
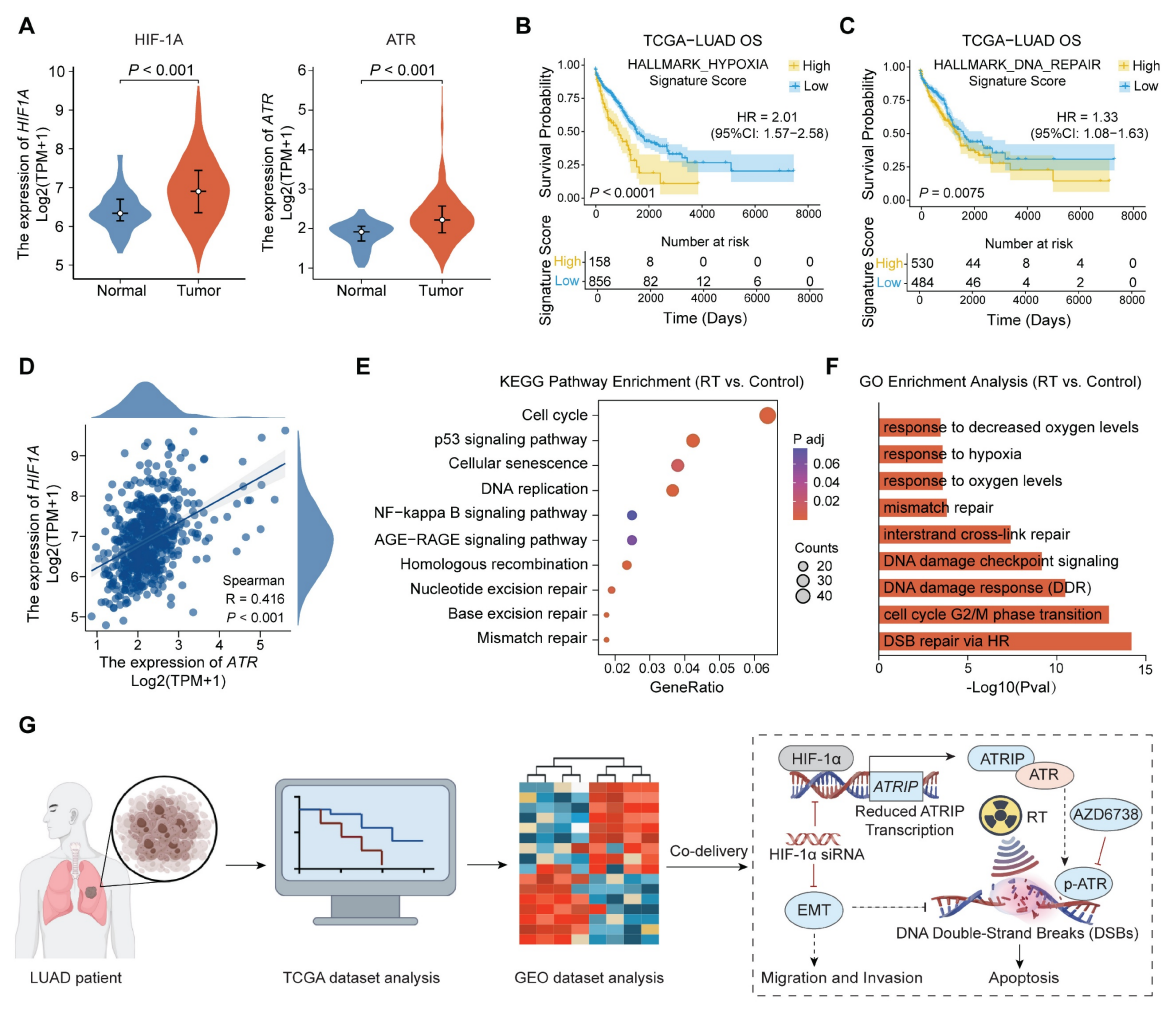
** Clinical relevance of the HIF-1α/ATR axis in LUAD and the rationale for dual-targeting radiosensitization.** (A) Differential mRNA expression of *HIF-1α* and *ATR* in LUAD and adjacent normal tissues from TCGA cohort. (B, C) Overall survival (OS) of patients stratified by high versus low expression of (B) HALLMARK_HYPOXIA and (C) HALLMARK_DNA_REPAIR gene signatures using the median value as a cut-off, with hazard ratios (HRs) and 95% confidence intervals (CIs) estimated using Cox proportional hazards models. (D) Spearman correlation analysis between *HIF-1α* and* ATR* mRNA levels in LUAD tumors. (E, F) Enrichment analyses were performed on DEGs identified between irradiated and non-irradiated LUAD samples in the GEO dataset GSE239495, including (E) KEGG pathway analysis and (F) GO biological process analysis. (G) Schematic illustration of integrating clinical transcriptomic data from TCGA and radiation-response transcriptomic data from GEO to reveal the interplay between hypoxia and ATR-associated signaling and guide the design of a co-delivery nanotherapeutic strategy for radiosensitization.

To assess the clinical relevance of a hypoxic microenvironment and the level of DDR in LUAD, we performed differential expression analysis using patient tumor samples from TCGA. As shown in Figure 1A, the mRNA expression levels of both *HIF-1α* and *ATR* are significantly elevated in LUAD tissues compared to those in adjacent normal tissues. Consistent with this observation, Kaplan-Meier survival analysis confirms that higher scores for the HALLMARK_HYPOXIA and HALLMARK_DNA_REPAIR gene signatures are associated with poorer overall survival (OS) in LUAD patients (Figure 1B, C). Cox proportional hazards analysis further supports these associations, yielding hazard ratios (HRs) of 2.01 (95% CI, 1.57-2.58) for HALLMARK_HYPOXIA and 1.33 (95% CI, 1.08-1.63) for HALLMARK_DNA_REPAIR. Moreover, Spearman correlation analysis showed that HIF-1α and ATR mRNA expression levels were strongly positively correlated in LUAD tumors (Figure 1D), suggesting coordinated activation of hypoxia-associated programs and DDR signaling in clinical specimens, which may collectively support tumor survival under genotoxic stress.

Given the upregulation of *HIF-1α* and *ATR* in LUAD, we hypothesized that RT might reinforce these pro-survival programs by aggravating hypoxic adaptation and activating DNA repair pathways. To test this hypothesis, we analyzed differentially expressed genes (DEGs) in a Gene Expression Omnibus (GEO) dataset (GSE239495) comprising LUAD transcriptomes collected before and after irradiation. Kyoto Encyclopedia of Genes and Genomes (KEGG) and Gene Ontology (GO) enrichment analyses of post-irradiation samples reveal distinct activation of hypoxia-response programs, DDR-related pathways, and DNA replication/cell-cycle regulation (Figure 1E, F). These results indicate that irradiation induces an adaptive transcriptional response to strengthen HIF-1α- and ATR-associated signaling, thereby promoting the establishment of a radioresistant phenotype.

Together, these clinical and transcriptomic findings provide a strong rationale for simultaneously targeting hypoxia adaptation and ATR-mediated checkpoint protection to improve RT responsiveness in LUAD (Figure 1G). However, the intrinsic physicochemical disparity between nucleic acids (e.g., siRNA) and hydrophobic small-molecule inhibitors (e.g., ATR inhibitors) often hinders their coordinated delivery and simultaneous target engagement within tumors. To address this challenge, we developed a biocompatible and structurally tunable nanomedicine to allow spatiotemporally synchronized co-delivery of HIF-1α siRNA and an ATR inhibitor, thereby optimizing intratumoral drug exposure and maximizing synergistic therapeutic efficacy.

### Design, preparation, and biosafety of redox-responsive polymeric nanocarriers

**Figure 2:**
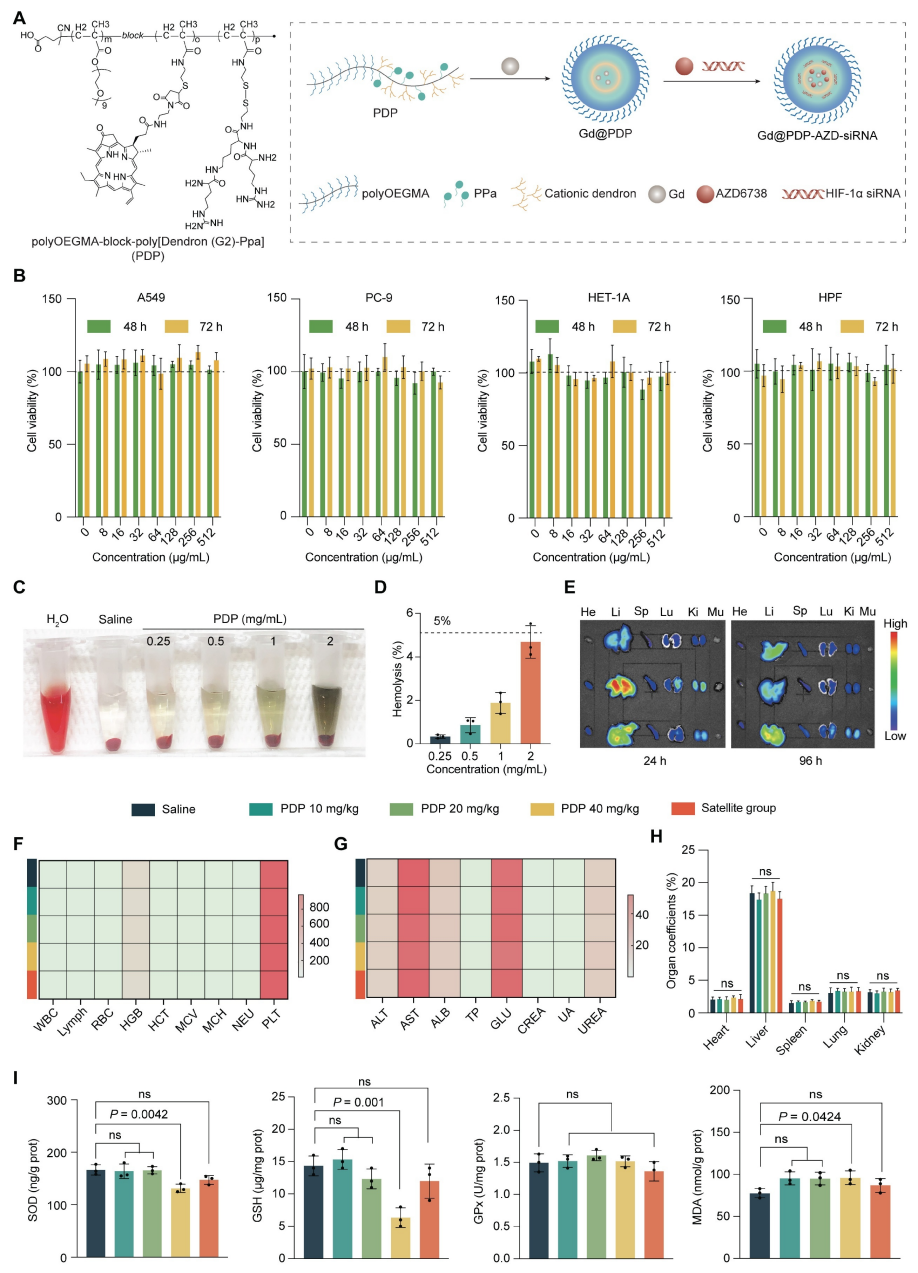
** Design and biosafety evaluation of PDP.** (A) Schematic illustration of the PDP multifunctional nanoplatform. (B) Cell viabilities of A549, PC-9, HET-1A, and HPF cells after incubation with PDP, with concentrations ranging up to 512 μg/mL, for 48 or 72 h via the CCK-8 assay (n = 5). (C) Representative images and (D) quantitative analysis of hemolysis induced by PDP at a concentration of 0.25, 0.5, 1.0 and 2.0 mg/mL (n = 3). (E) *Ex vivo* fluorescence images of major organs (He: heart; Li: liver; Sp: spleen; Lu: lungs; Ki: kidneys; Mu: muscles) from healthy mice at 24 h or 96 h after injection (n = 3). (F-I) Systemic safety profiles after a 28-day treatment and 14-day recovery in healthy male mice: (F) complete blood count (CBC) parameters; (G) serum biochemical markers for the hepatic and renal function; (H) organ coefficients; and (I) hepatic oxidative stress markers (SOD, GSH, GPx, MDA) (n = 5). Mean ± standard deviation (SD); ns: not significant.

To enable co-targeting of HIF-1α and ATR, we developed a redox-responsive polymeric nanocarrier as a multimodal radiosensitization platform (Figure 2A). Based on a previously reported dendritic polymer system 18, an amphiphilic block copolymer (PDP) was synthesized by reversible addition-fragmentation chain-transfer (RAFT) polymerization of hydrophilic poly(oligo(ethylene glycol) methyl ether methacrylate) (polyOEGMA), followed by modular conjugation of generation-2 (G2) cationic dendrons and pyropheophorbide a (Ppa) (Scheme S1-S2). Successful synthesis of intermediates and the final copolymer is confirmed by proton nuclear magnetic resonance spectroscopy (¹H NMR), liquid chromatography-mass spectrometry (LC-MS), and high-resolution mass spectrometry (HR-MS) (Figure S1-S14). A characteristic absorption peak at approximately 665 nm for Ppa in the UV-vis spectrum of PDP suggests successful Ppa conjugation. A loading Ppa content of 3.92 wt% in PDP (Figure S15, S16) supports its application in RDT.

The biosafety of PDP was systematically evaluated before its use for drug delivery and radiosensitization. PDP exhibits negligible *in vitro* cytotoxicity in both normal (HET-1A and HPFs) and LUAD (A549 and PC-9) cell lines, and there is no significant reduction in their viabilities after 48 or 72 h exposure at 0-512 μg/mL (Figure 2B). PDP also displays excellent hemocompatibility, and the hemolysis rate is below a safety threshold of 5% for the tested concentrations (Figure 2C, D).

In healthy mice, fluorescence imaging reveals that PDP is predominantly accumulated in the liver within 24-96 h following intravenous administration (Figure 2E and Figure S17). To assess systemic safety, fifty BALB/c mice (equal sex ratio) were randomized into five groups (n = 10 mice/group): a saline group, three PDP treatment groups (10, 20, and 40 mg/kg), and a high-dose satellite group for recovery assessment. Nanoparticles were administered every four days for 28 days. Main treatment groups were analyzed at the end of the dosing period, while the satellite group underwent a 14-day drug-free washout period before evaluation (Figure S18). Throughout the treatment and recovery phases, all animals exhibit normal clinical behavior, maintain stable body weights, and display no observable signs of systemic toxicity (Figure S19). Hematological indices, serum biochemical parameters, and organ coefficients remain within normal ranges (Figure 2F-H, Figure S20, and Table S2-S4). Histological analysis (H&E staining) of major organs reveals no treatment-related abnormalities (Figure S21). Although prolonged high-dose administration induces a transient reduction in hepatic SOD and GSH levels, these levels fully recover after the washout period (Figure 2I), indicating that the observed changes represent a reversible adaptive response rather than permanent oxidative injury.

Taken together, these results support that PDP could be used as a biocompatible and well-tolerated nanocarrier for co-delivery of HIF-1α siRNA and AZD6738 for LUAD radiosensitization.

### PDP achieves efficient intracellular siRNA delivery for functional HIF-1α silencing under hypoxia

**Figure 3:**
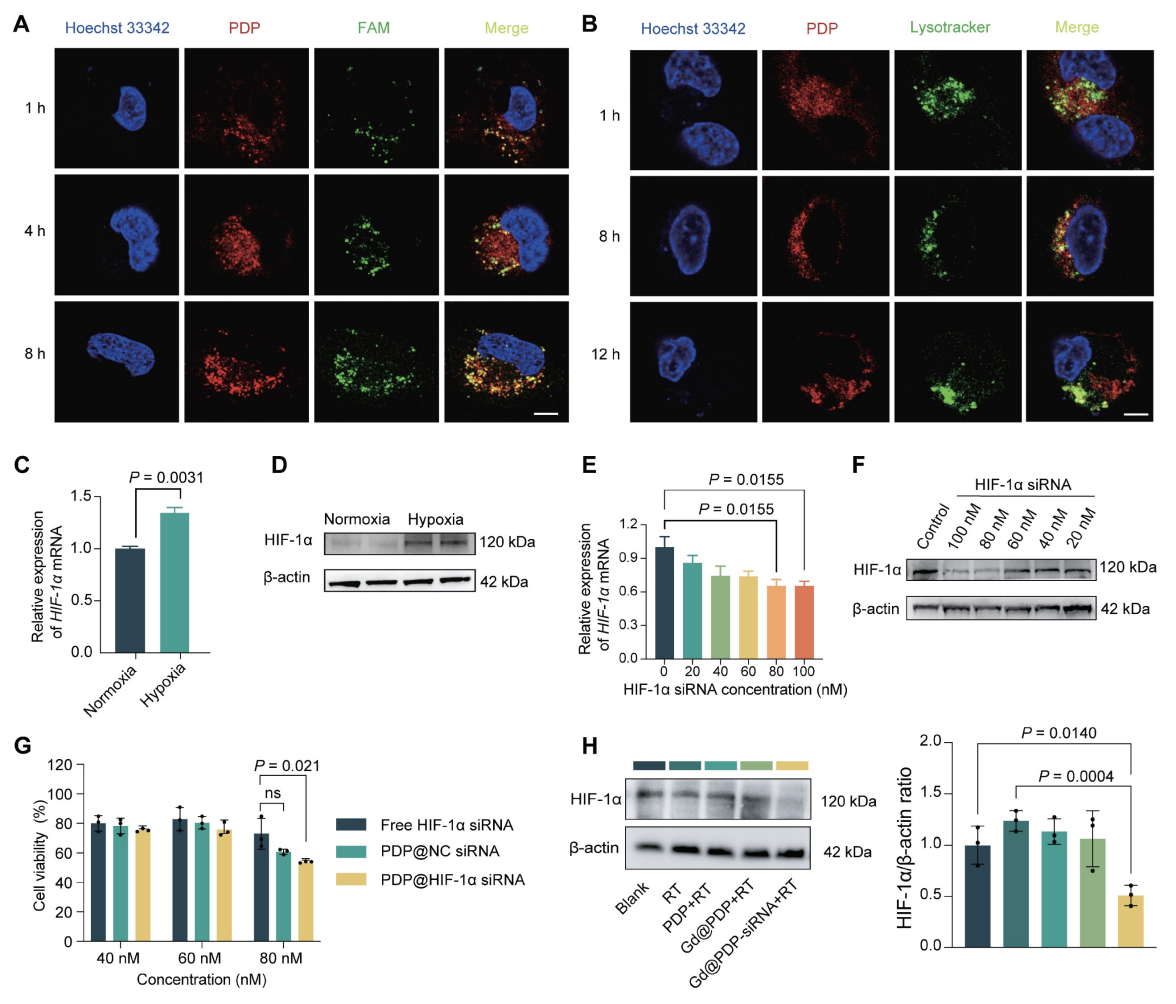
** Efficient intracellular siRNA delivery and hypoxia-responsive HIF-1α silencing mediated by the PDP nanocarrier.** (A) Confocal microscopic images for cellular uptake of PDP@FAM-siRNA by A549 cells (n = 3). Scale bar: 5 µm. (B) CLSM images for endo/lysosomal escape of PDP@NC-siRNA in A549 cells (n = 3). Scale bar: 5 µm. (C, D) HIF-1α mRNA (qRT-PCR) expression levels and protein concentrations (western blot) in A549 cells under normoxic or hypoxic conditions (n = 3). (E, F) The ability of PDP@HIF-1α siRNA to downregulate HIF-1α in a dose-dependent manner in A549 cells was evaluated by qRT-PCR (E) and western blotting (F) (n = 3). (G) Viabilities of A549 cells treated with free HIF-1α siRNA, PDP@NC-siRNA, or PDP@HIF-1α siRNA (n = 3). (H) HIF-1α protein levels in hypoxic A549 cells treated with different formulations with or without RT (n = 3). Mean ± SD; ns: not significant.

We first assessed intracellular FAM-labeled siRNA delivery by PDP. Confocal laser scanning microscopy (CLSM) images reveal time-dependent cellular uptake of PDP@FAM-siRNA by A549 cells (Figure 3A). Weak fluorescence signals are observed at 1 h, and the signal intensity is increased markedly at 4 h and further intensified at 8 h. These results confirm efficient siRNA loading and PDP-mediated intracellular accumulation. Given that effective endo/lysosomal escape is essential for preventing cargo degradation and ensuring cytosolic bioavailability, we next examined intracellular trafficking of PDP@NC-siRNA. At 8 h post-incubation, a high degree of co-localization of red signals (PDP@NC-siRNA) and green signals (LysoTracker for lysosomes) is observed, however, two signals are distinctively separated at 12 h, indicating successful endo/lysosomal escape of PDP@NC-siRNA (Figure 3B). Together, these findings demonstrate that PDP supports efficient cellular internalization and timely endosomal release, thereby realizing effective cytosolic siRNA delivery.

We next evaluated functional silencing of HIF-1α under hypoxic conditions after PDP-mediated delivery of siRNA. Hypoxic exposure (1% O₂) significantly increased HIF-1α transcription and protein abundance in A549 cells (Figure 3C, D). To determine the optimal siRNA dose, cells were treated with PDP loaded with HIF-1α siRNA at various concentration. Both qRT-PCR and western blot analyses show that siRNA at 80 nM displays the strongest silencing effect (Figure 3E, F), and this dose is selected for subsequent experiments. At 80 nM, PDP@HIF-1α siRNA induces a substantially greater reduction in the A549 cell viability compared to free siRNA or PDP@NC-siRNA, a negative-control formulation (Figure 3G), confirming that PDP enhances siRNA bioactivity through efficient intracellular delivery.

To closely mimic a radiotherapy setting, an irradiation-responsive derivative, Gd@PDP, was employed for delivery of HIF-1α siRNA, resulting in Gd@PDP-siRNA. After internalization of Gd@PDP-siRNA, HIF-1α expression levels under hypoxia were evaluated in the presence or absence of irradiation. RT alone induces a modest but non-significant increase in the expression level of HIF-1α protein, which confirms an adaptive stress response is generated by tumor cells after RT. In contrast, formulations containing siRNA effectively suppress the adaptive upregulation (Figure 3H). Collectively, these results demonstrate that the PDP platform achieves efficient intracellular siRNA delivery for functional HIF-1α silencing under hypoxia and simultaneous suppression of RT-induced adaptive HIF-1α upregulation, thereby establishing a critical prerequisite for subsequent radiosensitization.

### Construction and characterization of the Gd-chelated and dual drug-loaded nanoplatform

To confer irradiation responsiveness on the PDP delivery platform, Gd³⁺ was introduced as a high-Z component through coordination with the Ppa moieties. Briefly, Gd³⁺ ions were chelated into the PDP structure under a mildly acidic aqueous condition (pH 5.2-5.4), followed by dialysis and lyophilization to yield Gd@PDP (Scheme S3). The Gd loading in Gd@PDP is approximately 0.019 wt% via inductively coupled plasma mass spectrometry (ICP-MS). Transmission electron microscopy (TEM) images confirm that the formed nanoparticles are uniformly spherical and have a distinct core-shell structure (Figure S22A). Dynamic light scattering (DLS) analysis shows that Gd@PDP has a hydrodynamic diameter of 85.1 ± 1.7 nm and a zeta potential of +19.6 mV (Figure S22B, C; Table S1), indicating successful formation of well-defined polymeric micelles with a moderately cationic surface.

This Gd@PDP platform was assessed for *in vivo* delivery and therapeutic application. A key function of the platform is to enhance irradiation-triggered ROS generation. We assessed its radiodynamic performance using a singlet oxygen probe, ABDA. As shown in Figure S23, the PDP polymer alone causes negligible decay in ABDA absorbance under X-ray irradiation (0-8 Gy), indicating the polymer has minimal intrinsic radiodynamic activity. In contrast, Gd@PDP induces a pronounced dose-dependent decrease in ABDA absorbance, indicating singlet oxygen generation is markedly enhanced. Together, these data support that Gd@PDP integrates intracellular cargo delivery with X-ray-activated RDT, thereby enabling synergistic ROS amplification during delivery of HIF-1α siRNA and AZD6738 for radiosensitization.

After the Gd@PDP platform was established, we next constructed the final therapeutic nanomedicine by co-loading AZD6738, an ATR inhibitor, and HIF-1α siRNA. AZD6738, a hydrophobic small-molecule drug, was first encapsulated into the hydrophobic core of Gd@PDP by thin-film hydration to generate Gd@PDP-AZD. DLS analysis reveals a hydrodynamic diameter of 83.3 ± 1.1 nm, which is comparable to that of the empty carrier, indicating that drug encapsulation does not compromise the nanoparticle integrity (Figure S22B, C; Table S1). According to High-performance liquid chromatography (HPLC) quantification, when the carrier-to-drug mass ratio was optimized to 40:1, Gd@PDP-AZD achieved a drug loading content (LC) of 2.14% and an encapsulation efficiency (EE) of 89.44%. To additionally incorporate a nucleic acid cargo, Gd@PDP-AZD-siRNA was prepared by electrostatic complexation of negatively charged HIF-1α siRNA with cationic Gd@PDP-AZD. Gel retardation analysis confirms complete siRNA complexation at a polymer-to-siRNA mass ratio of 200:1, which was kept for the subsequent experiments (Figure S24). The final Gd@PDP-AZD-siRNA nanocomplex retains a uniform spherical morphology and a stable hydrodynamic diameter of 83.2 ± 2.4 nm (Figure S22; Table S1), indicating successful integration of three functional components without aggregation. In addition, the hydrodynamic diameter of Gd@PDP-AZD-siRNA remains consistent over 48 h in various media (water, PBS, 1% FBS, and 10% FBS) at room temperature, as well as during 17 days of storage at 4 °C, confirming the excellent colloidal stability of the nanomedicine (Figure S25).

We examined TME-responsive release of siRNA from the nanocomplex under a reductive condition that mimics an elevated intracellular GSH level in tumor cells. In the absence of dithiothreitol (DTT), siRNA is tightly complexed with the Gd@PDP-AZD nanoparticles. By contrast, DTT at a concentration of 5-20 mM effectively triggers siRNA release from the nanocomplex (Figure S26). These findings demonstrate the nanomedicine displays great stability under a non-reducing condition, but shows a pronounced redox-responsive siRNA release profile under a reductive condition.

### Integrated radiodynamic ROS amplification and hypoxia-DDR disruption synergistically potentiate radiosensitization

**Figure 4:**
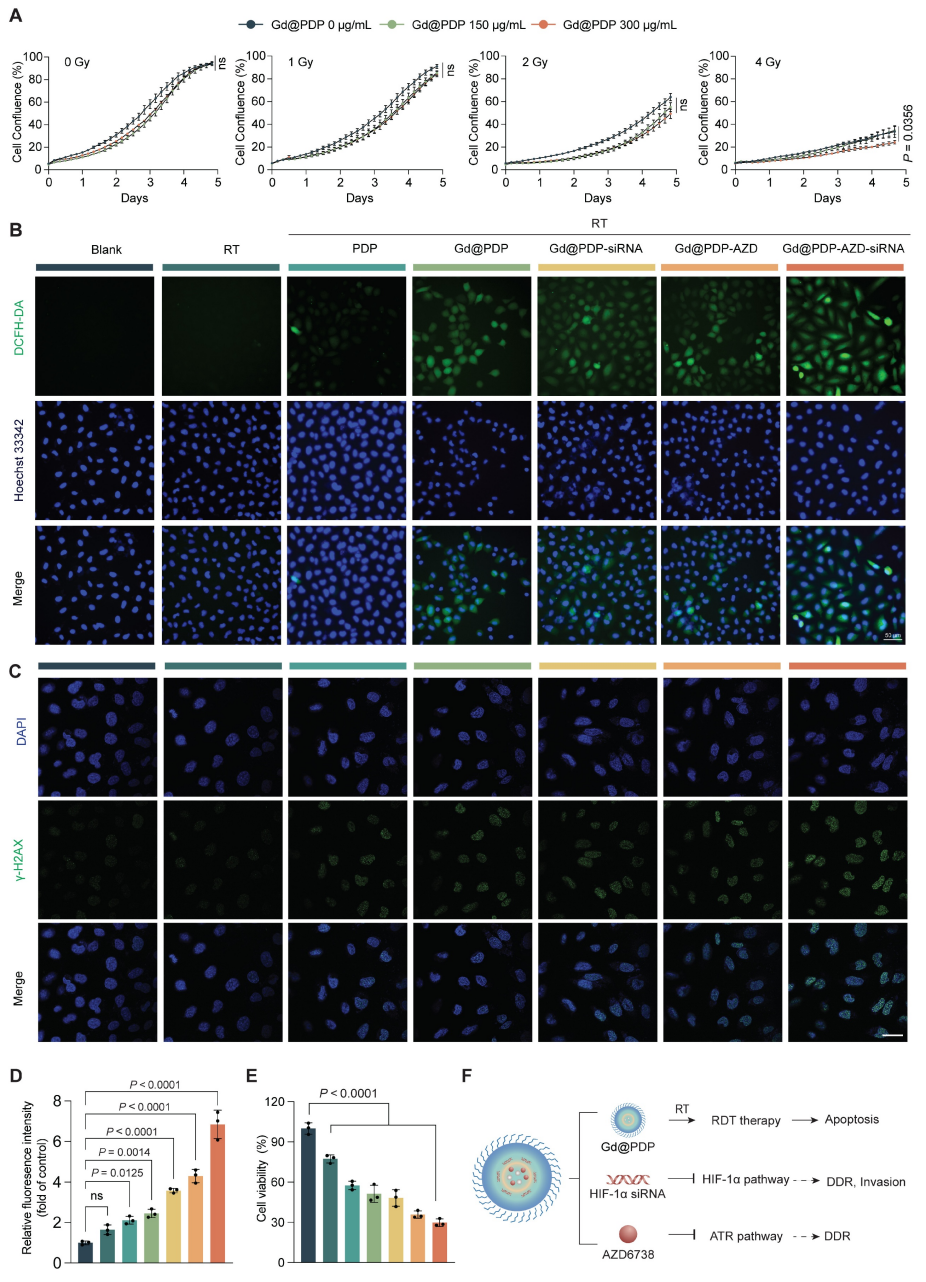
**Radiosensitization through integrated radiodynamic enhancement and hypoxia-DDR blockade.** (A) Real-time monitoring of the confluence level of H1975 cells treated with Gd@PDP and X-ray at a dose of 0-4 Gy via live-cell imaging (n = 3). (B) Representative DCFH-DA fluorescence images of intracellular ROS generation in A549 cells after treatment with the indicated formulations followed by 4 Gy X-ray irradiation. Scale bar: 50 µm. (C) Representative immunofluorescence images and (D) quantitative analysis of the γ-H2AX foci in A549 cells post-treatment (n = 3). Scale bar: 50 µm. (E) Viabilities of A549 cells exposed to different treatments combined with 4 Gy RT (n = 3). (F) Schematic diagram for the proposed intracellular synergistic mechanism. Mean ± SD; ns: not significant.

To confirm the function of the Gd-Ppa radiodynamic module, we first assessed the irradiation-responsive effect of the core carrier (Gd@PDP) via real-time live-cell monitoring. H1975 cells were treated with Gd@PDP (0, 150, and 300 μg/mL) and exposed to graded X-ray doses (0, 1, 2, and 4 Gy), and cell confluence levels were continuously monitored over 5 days. In the absence of irradiation, Gd@PDP exerts negligible effects on cell growth, supporting its no/low intrinsic cytotoxicity. Upon irradiation, however, Gd@PDP exposure results in dose-dependent suppression of cell growth, and the strongest inhibition level is observed at a radiation dose of 4 Gy and a Gd@PDP concentration of 300 μg/mL (Figure 4A), confirming functional performance of the radiodynamic module. Analysis of intracellular ROS generation confirms the result. Treatment with RT alone or PDP + RT induces marginal intracellular ROS generation, whereas treatment with Gd@PDP + RT triggers a marked increase in ROS levels (Figure 4B, Figure S27), consistent with the cell-free ABDA assay (Figure S23). The Gd@PDP-AZD-siRNA + RT group exhibits the highest ROS level, suggesting that the integrated nanoplatform could further amplify irradiation-induced oxidative damage 19. Notably, the levels of key endogenous antioxidants were not significantly different among all treatment groups (Figure S28), indicating that the observed ROS elevation primarily arises from enhanced radiodynamic activity rather than antioxidant impairment.

We examined DNA damage induced by ROS amplification through Gd@PDP-AZD-siRNA incubation and radiation. γ-H2AX immunofluorescence images reveal that Gd@PDP-AZD-siRNA + RT induces the strongest DNA damage response among all treatment groups (Figure 4C, D). Although irradiation results in an increase in the γ-H2AX foci broadly, the most intensive and widespread signals are observed in the combined formulation treatment group, indicating substantial accumulation of unrepaired DNA lesions after treatment with the nanomedicine.

To determine disruption of the hypoxia-DDR axis simultaneously, we analyzed ATR-Chk1 checkpoint signaling. Irradiation results in a marked increase in p-Chk1 (Ser345), consistent with activation of the replication stress checkpoint. HIF-1α silencing via Gd@PDP-siRNA significantly reduces RT-induced Chk1 phosphorylation, and AZD6738-containing formulations (Gd@PDP-AZD and Gd@PDP-AZD-siRNA) also suppress p-Chk1. The strongest inhibition of p-Chk1 is observed in the Gd@PDP-AZD-siRNA group. By contrast, there is a modest and non-significant decrease in p-ATR in the Gd@PDP-siRNA group, and the levels of total ATR and total Chk1 are barely changed (Figure S29). These findings indicate that HIF-1α silencing and ATR inhibition suppress checkpoint signaling primarily at the level of Chk1 phosphorylation. The impairment of DNA damage repair by Gd@PDP-AZD-siRNA is further corroborated by the most pronounced reduction in A549 cell viability (Figure 4E). Together, these results support a dual-strike radiosensitization mechanism for Gd@PDP-AZD-siRNA: initial damage induced by Gd-amplified radiodynamic ROS; followed by a reduction in damage tolerance and repair capacity through concurrent disruption of the hypoxia-DDR axis (Figure 4F).

### Gd@PDP-AZD-siRNA induces apoptosis and suppresses clonogenic survival and invasion

**Figure 5:**
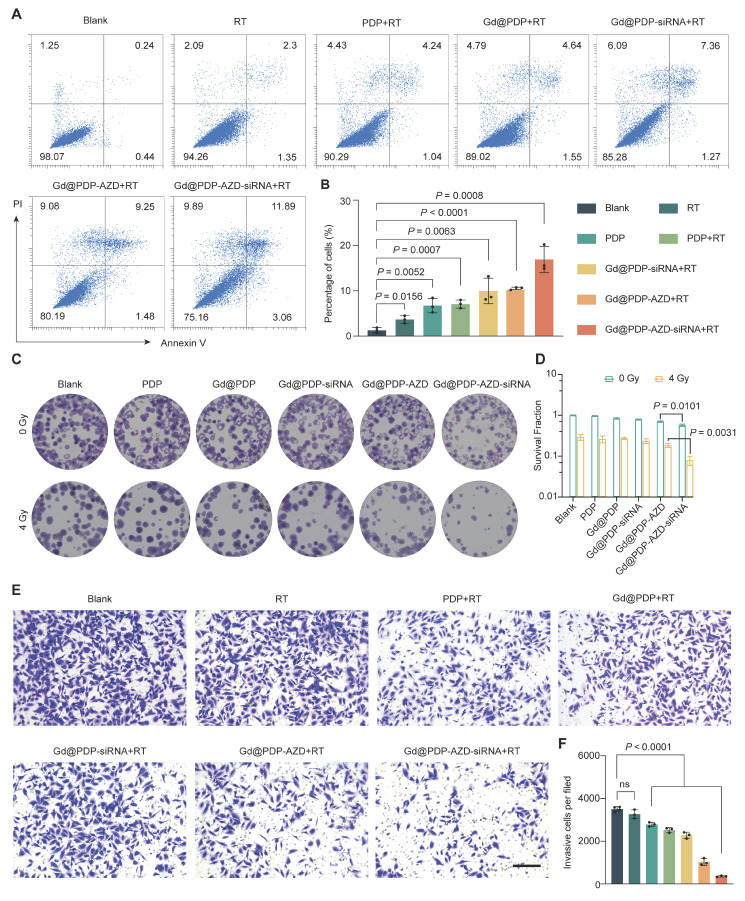
**Gd@PDP-AZD-siRNA enhances radiosensitivity by promoting apoptosis and suppressing clonogenic survival and invasion in LUAD cells.** (A) Representative Annexin V/PI flow cytometry plots of A549 cells following the indicated treatments combined with 4 Gy RT. (B) Quantitative analysis of the apoptotic rate from (A) (n = 3). (C) Representative images and (D) quantification of long-term clonogenic survival (n = 3) via the colony formation assay. (E) Representative images and (F) quantification of invasive cells via the Transwell invasion assay (n = 3). scale bar: 200 μm. Mean ± SD; ns: not significant.

Inspired by extensive molecular damage induced by the combination therapy, we examined its effects on key malignant phenotypes. Consistent with a marked increase in DNA damage and oxidative stress, the combined treatment triggers robust activation of apoptosis 20. RT alone induces a low level of apoptosis (~3.67%), while the addition of the nanomedicine results in an apoptotic rate of ~16.9% (Figure 5A, B), indicating that the RT-induced molecular lesions are sufficient to drive programmed cell death.

We next assessed whether the acute cytotoxicity could be translated into durable suppression of long-term proliferation of tumor cells. Clonogenic assays show that Gd@PDP-AZD-siRNA displays measurable antiproliferative activity and reduces colony formation in a concentration-dependent manner, which may be ascribed to the constitutive effects of ATR inhibition and HIF-1α silencing. Following exposure to 4 Gy irradiation, treatment with Gd@PDP-AZD-siRNA almost completely abolished clonogenic growth, reducing the relative surviving fraction to 0.079, whereas the control group remained at 1.0 (Figure 5C, D). These data indicate that the treatment not only efficiently eliminates tumor cells but also profoundly impairs their long-term reproduction, a key determinant of curative radiotherapy efficacy.

Finally, we evaluated the effect of this multimodal treatment on the invasive phenotype of tumor cells. Transwell invasion assays confirm that neither RT alone nor the empty carrier significantly inhibits tumor cell invasion. In contrast, treatment with siRNA-containing formulations markedly decreased the number of invading cells, and the strongest inhibitory effect was observed in the Gd@PDP-AZD-siRNA + RT group (Figure 5E, F). The pronounced anti-invasive activity may be ascribed to effective HIF-1α silencing, indicating that the nanoplatform not only enhances local tumor cell killing but also suppresses pathways associated with malignant progression and metastatic dissemination.

### *In vivo* tumor targeting and radiosensitizing efficacy in a clinically relevant LUAD PDX model

**Figure 6:**
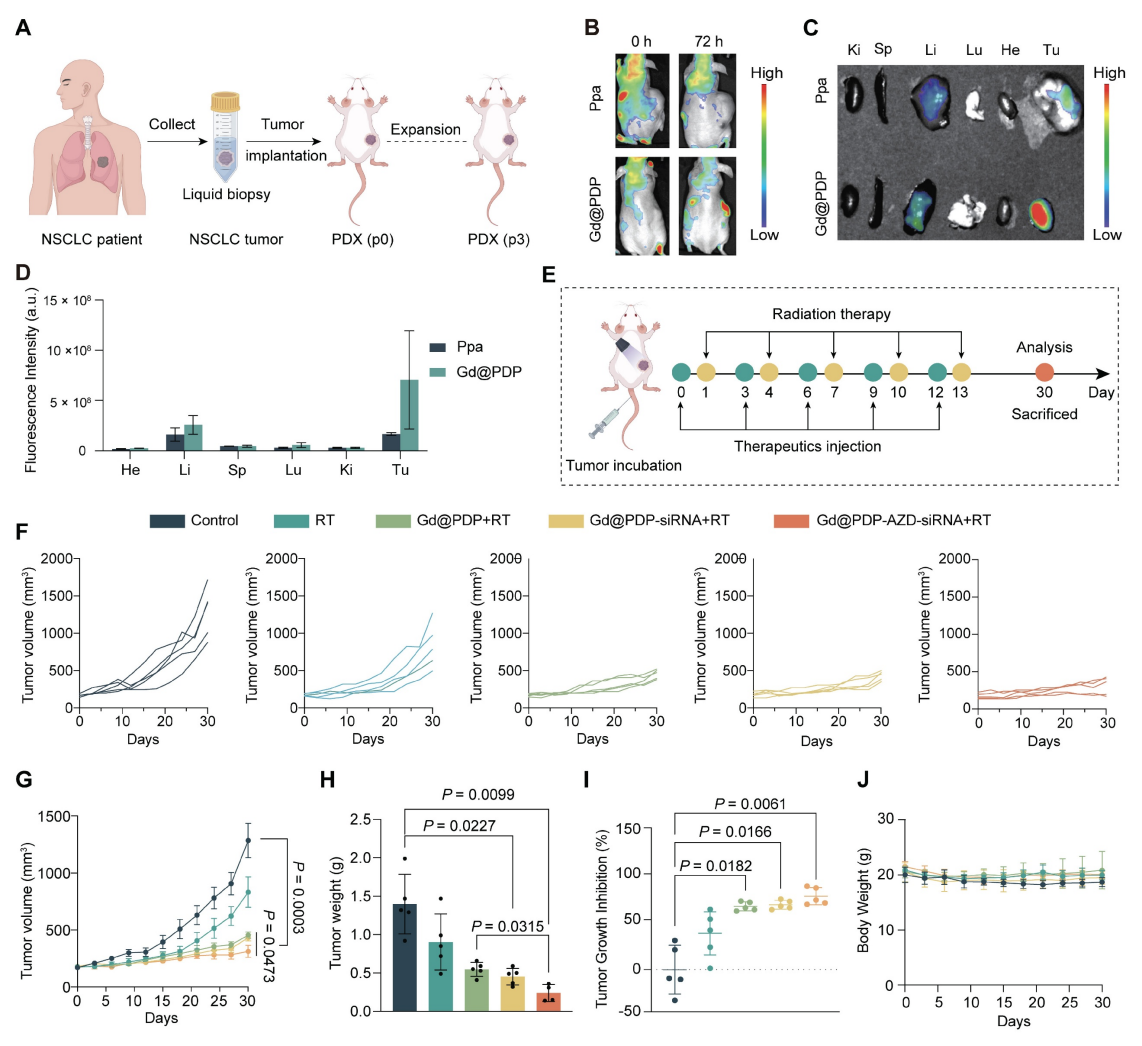
***In vivo* tumor targeting and therapeutic efficacy of the nanoformulation in a LUAD-PDX model.** (A) Schematic illustration of LUAD PDX model establishment. (B-D) Tumor accumulation of free pyropheophorbide a (Ppa) and Gd@PDP, assessed by (B) *in vivo* fluorescence imaging, (C) *ex vivo* fluorescence imaging, and (D) corresponding quantitative analysis (n = 2). Tu, tumor; Ki, kidneys; Lu, lungs; Sp, spleen; Li, liver; He, heart. (E) Schematic illustration of the treatment schedule for RT and nanomedicine administration. (F-G) Tumor growth curves in different treatment groups (saline, RT alone, Gd@PDP + RT, Gd@PDP-siRNA + RT, and Gd@PDP-AZD-siRNA + RT) (n = 5). (H) Tumor weights at the study endpoint (n = 5). (I) Tumor growth inhibition (TGI) for each treatment group (n = 5). (J) Body weight changes during the treatment period (n = 5). Mean ± SD; ns: not significant.

To validate our *in vitro* findings in a clinically relevant setting, we evaluated the therapeutic strategy in a LUAD PDX model (Figure 6A). PDX tumors were established in athymic nude mice and maintained at a low passage (P3) to minimize the passaging-induced drift. *In vivo* fluorescence imaging reveals that Gd@PDP efficiently accumulates and retains in tumor tissue at 72 h post-injection, whereas only weak and transient tumor fluorescence signals are observed in the group treated with free Ppa (Figure 6B-D). *Ex vivo* organ imaging confirms the tumor enrichment of the PDP formulation, alongside hepatic accumulation (Figure S30), supporting the favorable tumor-targeting profile of the nanomedicine.

When tumor volume reached approximately 150-250 mm³, the mice were randomly assigned into five groups (n = 5 each). Baseline tumor burden was well balanced across groups, with no significant difference observed before treatment initiation (*p* = 0.9847). The animals then received the indicated formulations according to the regimen illustrated in Figure 6E. RT alone produces modest tumor inhibition, while nanomedicine-containing regimens improve therapeutic efficacy to varying extents (Figure 6F-I and Figure S31). Among them, Gd@PDP-AZD-siRNA + RT results in the strongest and most sustained tumor suppression. A tumor growth inhibition (TGI) of 75.7% is achieved and tumor progression is completely arrested at the treatment endpoint (Figure 6I). This group also has the lowest terminal tumor weight (Figure 6H), confirming superior *in vivo* antitumor activity of the nanomedicine.

The combination regimen is well tolerated, with no treatment-related deaths, no observable behavioral abnormalities, and a stable body weight throughout the study period (Figure 6J). Furthermore, complete blood counts (red blood cells, white blood cells, platelets, and hemoglobin) and serum markers of hepatic function (aspartate aminotransferase, alanine aminotransferase, and albumin) are within their normal physiological ranges (Figure S32). It is observed that urea levels are significantly elevated in the RT-alone group, but remain within the normal range in the nanoformulation/RT-treated groups, supporting a favorable safety profile of the combination therapy.

### Mechanistic validation of *in vivo* radiosensitization

**Figure 7:**
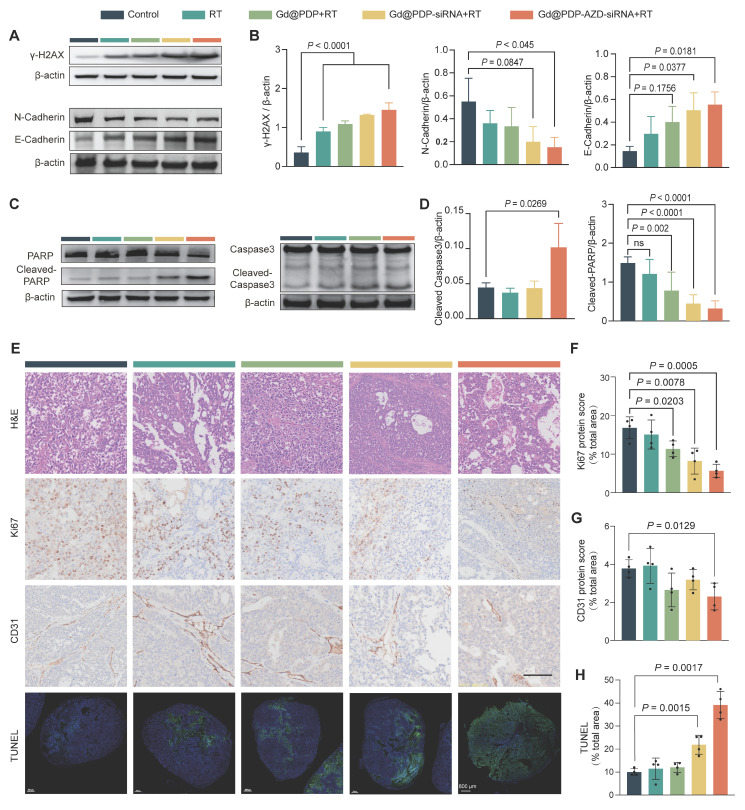
** Mechanistic validation in PDX tumors.** (A, B) Representative western blots (A) and corresponding quantitative analysis (B) of DNA damage (γ-H2AX) and EMT markers (n = 3). (C, D) Representative western blots (C) and corresponding quantification (D) of apoptosis markers (cleaved PARP and cleaved Caspase-3) (n = 3). (E) Histopathological and immunohistochemical analysis of tumor sections: H&E staining; immunohistochemistry (IHC) for proliferation (Ki-67) and angiogenesis (CD31); immunofluorescence (IF) for apoptosis (TUNEL). Scale bars: 100 μm (H&E, IHC); 800 μm (IF). (F-H) Quantification of (F) Ki-67, (G) CD31, and (H) TUNEL signals (n = 4). Mean ± SD; ns: not significant.

To elucidate the molecular mechanism underlying the superior *in vivo* therapeutic efficacy, we performed comprehensive analysis of harvested tumor tissues. The strongest γ-H2AX signals are seen in the tumors from the Gd@PDP-AZD-siRNA + RT group, which is consistent with the *in vitro* findings, supporting enhanced DNA damage *in vivo* (Figure 7A, B). In addition, apoptotic signaling is markedly activated, as evidenced by the elevated expression levels of cleaved Caspase-3 and cleaved PARP (c-PARP) (Figure 7C, D). Notably, an increase in the γ-H2AX signals is accompanied by elevated expression levels of cleaved Caspase-3 and c-PARP, indicating that enhanced DNA damage is closely associated with intensified apoptotic signaling *in vivo*. These molecular alterations are also corroborated by histopathological analyses. Ki-67 staining shows a treatment-dependent reduction in the proliferative activity, and the most pronounced suppression is observed in the Gd@PDP-AZD-siRNA + RT group (Figure 7E, F). Consistently, TUNEL staining reveals a progressive increase in the apoptotic cell population for all treatment groups, and the highest level of apoptosis is seen in the combination treatment group (Figure 7E, H). Together, the changes in the Ki-67 and TUNEL signals provide robust tissue-level evidence of potent proliferation inhibition and enhanced apoptosis *in vivo*.

In addition, CD31 signals are significantly reduced in the Gd@PDP-AZD-siRNA + RT group, suggesting suppression of tumor angiogenesis, consistent with effective blockade of hypoxia-driven pro-angiogenic signaling (Figure 7E, G). Moreover, inhibition of hypoxia-associated signaling attenuates epithelial-mesenchymal transition (EMT) *in vivo*, which is supported by restoration of E-cadherin and simultaneous downregulation of N-cadherin in the Gd@PDP-AZD-siRNA + RT group (Figure 7A, B). These EMT-associated changes are consistent with the *in vitro* invasion data, supporting a reduction in the invasiveness of LUAD tumors after HIF-1α pathway suppression. Collectively, these mutually reinforcing tissue-level alterations are induced by the multimodal radiosensitization strategy integrating X-ray-activated ROS amplification with the suppression of hypoxia-adaptive signaling and blockade of the ATR-mediated checkpoint pathway.

## Discussion

RT remains a mainstay for LUAD treatment, but its long-term efficacy is often limited due to adaptive survival programs in tumor cells that are reinforced under therapeutic stress to ultimately develop radioresistance. Hypoxia and enhanced DDR are two closely interconnected and well-recognized mediators in adaptive survival programs 21. Herein, we discover a clinically relevant interplay between hypoxia-driven adaptation and ATR-mediated replication stress checkpoint in LUAD. Analysis of the TCGA-LUAD cohort reveals significant co-upregulation of *HIF-1α* and *ATR*, and there is a strong positive correlation between their expression levels, suggesting that hypoxia-associated adaptation and ATR-dependent stress tolerance may simultaneously occur and cooperatively contribute to tumor survival in patients. Consistently, elevated hypoxia and high DNA repair signature scores are associated with poor OS, confirming the prognostic relevance of this adaptive state. In addition, transcriptomic analysis of paired pre- and post-irradiation samples indicates that RT can strengthen these tolerance programs, evidenced by post-treatment enrichment in hypoxia-response, DNA repair, and cell-cycle-associated pathways. These findings support that irradiation intensifies hypoxia and replication stress, thereby reinforcing ATR-dependent checkpoint activation and repair engagement to establish a self-sustaining radioresistant mechanism. This mechanism may explain why RT alone or single-target interventions against either hypoxia or DDR often fails to achieve durable responses, and it provides a compelling rationale for multimodal strategies to concurrently disrupt hypoxia adaptation and ATR-mediated tolerance.

Based on this rationale, we developed a dual-targeting strategy to direct against HIF-1α, a master regulator of hypoxia adaptation, and ATR, a key kinase in the replication stress checkpoint. HIF-1α siRNA suppresses hypoxia-associated pro-survival transcriptional programs, while ATR inhibition facilitates escape of checkpoint protection and weakens replication stress tolerance. In combination, these interventions are designed to shift RT-induced DNA lesions from a repairable, buffered state into a lethal, irreparable damage one. A major translational challenge, however, lies in distinct physicochemical and pharmacokinetic properties of siRNA and hydrophobic small-molecule inhibitors, which lead to asynchronous tumor exposure and inefficient co-targeting in tumor cells 22. To overcome this challenge, we engineered a biocompatible, structurally tunable polymeric nanocarrier to achieve co-delivery of both agents with spatiotemporally coordinated intratumoral exposure, thereby maximizing synergistic radiosensitization. Mechanistically, this strategy is distinct from conventional approaches utilizing DDR-targeted radiosensitizers, such as PARP inhibitors (PARPi), or physical dose enhancement. While PARPi predominantly act through PARP-dependent repair blockade and PARP-trapping-associated replication stress, our platform targets ATR-mediated checkpoint defense and simultaneously suppresses HIF-1α-driven hypoxia adaptation. This approach disrupts an RT-reinforced tolerance network rather than perturbing a single repair pathway 23. Unlike conventional hypoxia modifiers that primarily target the hypoxic TME alone, our platform integrates hypoxia-targeted intervention with ATR checkpoint inhibition and Gd-enabled radiodynamic amplification within a single nanoplatform. Therefore, our design is well aligned with emerging integrated strategies by combining amplification of cellular damage with disruption of adaptive tolerance programs, thereby biasing RT-induced lesions toward persistence and lethality.

Polymeric nanocarriers have become an important platform in oncology, and several clinically approved formulations have displayed improved therapeutic indices through enhanced tumor accumulation and reduced systemic toxicity 24-28. Building on our previously reported dendritic polymer system 18, we optimized an amphiphilic block copolymer (PDP) for dual-payload co-delivery. Its cationic G2 dendritic domain allows efficient electrostatic complexing with HIF-1α siRNA, and its amphiphilic architecture facilitates hydrophobic encapsulation of AZD6738. Covalent incorporation of Ppa in PDP retains its near-infrared absorption at approximately 665 nm, thereby providing the structural basis for X-ray-activated RDT. Favorable *in vitro* and *in vivo* tolerability of PDP establishes it as a feasible co-delivery scaffold for multimodal radiosensitization 29-31. Efficient intracellular delivery and controlled subcellular release of dual payloads from the nanoplatform are central to the therapeutic synergy. Rapid cellular uptake and efficient endo/lysosomal escape of siRNA and AZD6738 in the PDP nanocarrier are obtained in this study, and endo/lysosomal escape may be credited to the proton sponge effect of the arginine-rich dendritic domain in PDP 18, 32, 33. In addition, a redox-responsive, disulfide-linked architecture in the nanomedicine provides its extracellular stability while promoting rapid payload release in a reducing cytosolic environment in tumor cells. Functional HIF-1α silencing under hypoxia, together with the associated antiproliferative effect in A549 cells, supports that released siRNA through PDP-mediated delivery successfully suppresses HIF-1α-dependent survival and metabolic programs and molecular gene silencing leads to inhibition of biological activity in cancer cells 34, 35.

Beyond biological pathway inhibition, another important strategy for radiosensitization is to amplify cytotoxic ROS generation during RT. In this context, we incorporated high-Z Gd³⁺ into the PDP scaffold through coordination with Ppa, thereby creating a locally enriched radiosensitizing microenvironment for enhanced energy deposition. Under X-ray irradiation, Gd@PDP markedly enhances radiodynamic ROS generation, which is accompanied by inhibited tumor cell growth. Localized delivery and sustained release of high-Z Gd³⁺ from a nanocarrier can achieve effective radiosensitization and minimize the risk of long-term Gd³⁺ accumulation. A salient feature of the radiosensitization observed in this study is that it originates from the coordinated modulation of both damage generation and damage fate, rather than from a singular mechanism. Standalone high-Z sensitizers, including clinically used formulations such as hafnium oxide nanoparticles (Hensify®/NBTXR3) and ultrasmall Gd-based AGuIX, primarily enhance local energy deposition and/or ROS production, but do not directly interact with hypoxia-adaptive transcriptional programs or checkpoint-mediated tolerance mechanisms that buffer RT-induced lesions. Several high-Z platforms for multi-module radiosensitization have been engineered to integrate biological functions beyond dose deposition, such as RT-RDT systems with checkpoint-blockade potentiation 36, oxidative stress-driven enhancement of immunogenic cell death (ICD) 37, and strategies coupling energy deposition with DNA repair inhibition or suppression of hypoxia-associated signaling to bolster RT responses 38, 39. In the present study, a coordinated strategy of damage amplification coupled with repair/tolerance disruption is realized via Gd@PDP-AZD-siRNA. Within this nanomedicine, Gd-enabled RDT increases the initial damage burden, while HIF-1α silencing and ATR inhibition suppress hypoxia-adaptive survival and checkpoint-mediated tolerance. *In vitro* data support that the triple-combination treatment induces sustained γ-H2AX accumulation, markedly impairs clonogenic survival, and reduces the invasive capacity, supporting a shift in the fate of the RT-induced lesions from repair and recovery to persistence and cytotoxicity 35, 40-43.

This therapeutic concept was validated in a clinically relevant PDX model. Gd@PDP shows preferential tumor accumulation and prolonged retention *in vivo*, supporting spatially and temporally coordinated delivery of multicomponent payloads 44, 45. Importantly, Gd@PDP-AZD-siRNA + RT achieves robust tumor inhibition (~75.7% TGI) without significant systemic toxicity. Molecular and histological analyses consistently support a mechanism centered on damage amplification coupled with repair/tolerance disruption, evidenced by increased γ-H2AX, reduced Ki-67, and elevated TUNEL signals. In addition, reduced CD31 staining and reciprocal regulation of E-cadherin and N-cadherin confirm suppression of angiogenesis and attenuation of EMT. Although the levels of HIF-1α and VEGF are not directly measured in PDX tissues, their changes are consistent with effective disruption of hypoxia-adaptive signaling 8, 46, 47. Together, these findings confirm that the nanoplatform mediates synergistic radiosensitization through two interconnected mechanisms: enhanced radiodynamic DNA damage generation and concurrent impairment of repair and adaptive tolerance pathways.

Overall, our findings validate a mechanistically coordinated radiosensitization strategy for LUAD. This approach integrates physical amplification of radiation-induced damage via Gd-enhanced, X-ray-activated radiodynamic ROS generation with biological disruption of adaptive tolerance programs through HIF-1α silencing and ATR inhibition. The Gd@PDP-AZD-siRNA nanomedicine successfully achieves spatiotemporally coordinated co-delivery of siRNA and a hydrophobic ATR inhibitor, thereby addressing a significant challenge in combination nanomedicine design. In a clinically relevant PDX model, the nanomedicine achieves durable antitumor activity with favorable short-term tolerability. These proof-of-concept findings lay the foundation for continuing preclinical development of this multi-axis strategy to overcome adaptive radioresistance in LUAD. However, prior to clinical translation, several issues must be resolved. Scale-up and GMP manufacturing of Gd@PDP-AZD-siRNA will require stringent control over composition, loading efficiency, Gd/Ppa coordination, particle-size distribution, and release behavior. Furthermore, specifications for sterility, endotoxin levels, and storage stability must be validated during GMP production. Long-term toxicology studies, particularly under repeated-dose administration combined with radiotherapy, will be essential to evaluate chronic biodistribution, hepatic and splenic accumulation, potential Gd retention, immunotoxicity, and delayed organ injury. Finally, clinical development programs should be designed to optimize fractionated RT regimens and facilitate biomarker-guided patient selection. Early-phase clinical studies should incorporate pharmacokinetic/pharmacodynamic confirmation of intratumoral target engagement and schedule compatibility with standard radiotherapy. Addressing these issues can facilitate translation of this proof-of-concept into a clinically deployable radiosensitization platform.

## Conclusion

In summary, to overcome radioresistance driven by intertwined hypoxia signaling and ATR-mediated DDR in LUAD, we developed a redox-responsive, Gd-chelated polymeric nanoplatform for co-delivery of HIF-1α siRNA and the ATR inhibitor AZD6738. Two complementary actions are realized in this multimodal approach: (1) physical amplification of DNA damage via Gd-enhanced, X-ray-activated radiodynamic ROS generation, and (2) biological suppression of key adaptive resistance pathways via concurrent HIF-1α silencing and ATR checkpoint inhibition. Profound tumor suppression with an excellent biosafety profile is achieved in a clinically relevant PDX model. These encouraging therapeutic outcomes suggest our robust, synergistic, and translatable nanotherapeutic strategy could be applied for sensitizing LUAD to radiotherapy, offering a promising avenue for overcoming adaptive resistance in solid tumors.

## Materials and Methods

### Materials and characterization methods

Materials, synthesis and preparation of polymeric nanoparticles, and detailed characterization procedures are provided in the Supplementary Information (Methods, Sections S1-S2).

### Expression, prognostic relevance, and correlation analysis of *HIF-1α* and *ATR*

Using the TCGA-LUAD cohort, we compared the mRNA expression profiles of HIF1A and ATR between LUAD tumors and normal lung tissues. To assess the prognostic relevance of the pathway activity, LUAD patients were stratified separately into low- and high-score groups using median cut-off based on the Hallmark hypoxia signature (HALLMARK_HYPOXIA) and the Hallmark DNA repair signature (HALLMARK_DNA_REPAIR), with OS evaluated using Kaplan-Meier analysis and the log-rank test. Finally, the correlation between *HIF1A* and *ATR* expression levels in LUAD tumors was analyzed to generate the Spearman correlation coefficient and its corresponding *p*-value.

### Functional enrichment analysis

Bulk RNA sequencing (RNA-seq) data from the GSE239495 dataset were analyzed to identify DEGs between pre-irradiation and post-irradiation A549 cells. Differential expression analysis was performed using DESeq2, and genes above the predefined significance threshold were utilized for downstream analyses. Functional enrichment analyses were conducted based on the DEG list to identify major biological processes and signaling pathways associated with LUAD.

### Cell culture

HPFs were established from donor-derived lung tissues in-house and authenticated prior to experimentation. A549, PC-9, and H1975 human LUAD cells, along with HET-1A cells and HPFs, were cultured and maintained at 37 °C in a humidified incubator with 5% CO₂. When hypoxic culture was required, cells were incubated in a modular chamber equilibrated with a gas mixture consisting of 1% O_2_, 5% CO_2_, and balanced N_2_.

### Cellular uptake and intracellular trafficking

For cellular uptake studies, A549 cells were treated with PDP nanoparticles loaded with FAM-siRNA for 1, 4, or 8 h. After washing, the cells were counterstained with Hoechst 33342, and observed using a CLSM (Leica STELLARIS). To investigate endo/lysosomal escape, cells treated with PDP@NC-siRNA nanoparticles were stained with LysoTracker™ Green and Hoechst 33342 at predetermined time intervals and imaged by CLSM to assess colocalization.

### Cell viability and radiosensitivity assays

*Cytotoxicity*: Cytotoxicity of the PDP polymer was examined using the CCK-8 assay in A549, PC-9, HET-1A, and HPF cells. Cells were seeded into 96-well plates. After treatment, CCK-8 reagent was introduced into each well, and absorbance at 450 nm was recorded following 1-4 h of incubation.*Real-time proliferation monitoring*: H1975 cells were plated in 96-well plates and exposed to Gd@PDP for 6 h, followed by irradiation at doses of 0-4 Gy. The treated cells were then transferred to an Incucyte® live-cell analysis system, and cell confluence was recorded at 4 h intervals for a total of 5 days.

### *In vivo* biosafety assessment

To evaluate the systemic toxicity profile of PDP, fifty BALB/c mice with an equal sex distribution were randomly allocated into five groups: saline, PDP (10, 20, or 40 mg/kg), and a satellite group (PDP 40 mg/kg). Mice in the treatment groups received intravenous injections every 4 days for 28 days. The satellite group was observed for an additional 14-day drug-free period to assess the reversibility of treatment-related effects. Body weight and clinical signs were monitored throughout the study at 4-day intervals.

At the end of the experiment, blood was collected via retro-orbital puncture for complete blood counts and serum biochemistry analyses. Major organs (heart, kidneys, spleen, lungs, and liver) were harvested, weighed to calculate the organ coefficients, and processed for histopathological evaluation with H&E staining. Furthermore, liver tissues were specifically analyzed for markers of oxidative stress (SOD, GSH, GPx, MDA).

### Animals and the PDX model

The experimental animals (BALB/c nude and NOD-SCID mice, 6-8 weeks) were housed in an SPF-grade animal facility. The PDX model was established by subcutaneously implanting freshly resected lung adenocarcinoma tumor fragments into the right flank of NOD-SCID mice. When tumor volumes reached approximately 1000 mm³, the tumors were excised, sectioned into small fragments, and serially transplanted into BALB/c nude mice for subsequent experiments. All *in vivo* experiments were performed in compliance with protocols approved by Animal Ethics Committee of West China Hospital, Sichuan University.

### *In vivo* biodistribution

PDX-bearing nude mice received intravenous administration of either free Ppa or Gd@PDP at an equivalent Ppa dosage. Whole-body fluorescence signals were acquired at predetermined time points using a Kino near-infrared imaging system (Spectral Instruments Imaging, USA). In an independent experiment, healthy nude mice were treated with PDP nanoparticles to evaluate their long-term retention and tissue distribution. At the endpoint, major organs were collected for *ex vivo* fluorescence imaging.

### *In vivo* antitumor efficacy

PDX mice were randomly allocated into the following groups (n = 5 mice/group): (1) saline, (2) RT alone, (3) Gd@PDP + RT, (4) Gd@PDP-siRNA + RT, and (5) Gd@PDP-AZD-siRNA + RT. Nanoformulations were administered intravenously at 40 mg/kg every three days. Local tumor RT (3 Gy per fraction) was administered at 24 h after each nanoformulation injection using a small-animal irradiator. Tumor growth and body weight were recorded at 3-day intervals throughout the study. At day 30, tumors were excised and weighed, after which tumor tissues were collected for subsequent mechanistic analyses.

### Statistical analysis

Statistical analyses were performed using GraphPad Prism 8.0. Comparisons between two groups were conducted using an unpaired two-tailed Student's t-test. Comparisons among multiple groups were performed using one-way ANOVA followed by Tukey's or Dunnett's post hoc test. A p-value of less than 0.05 was considered statistically significant.

## Supplementary Material

Supplementary materials, instrumentations, methods, figures and tables.

## Figures and Tables

**Scheme 1:**
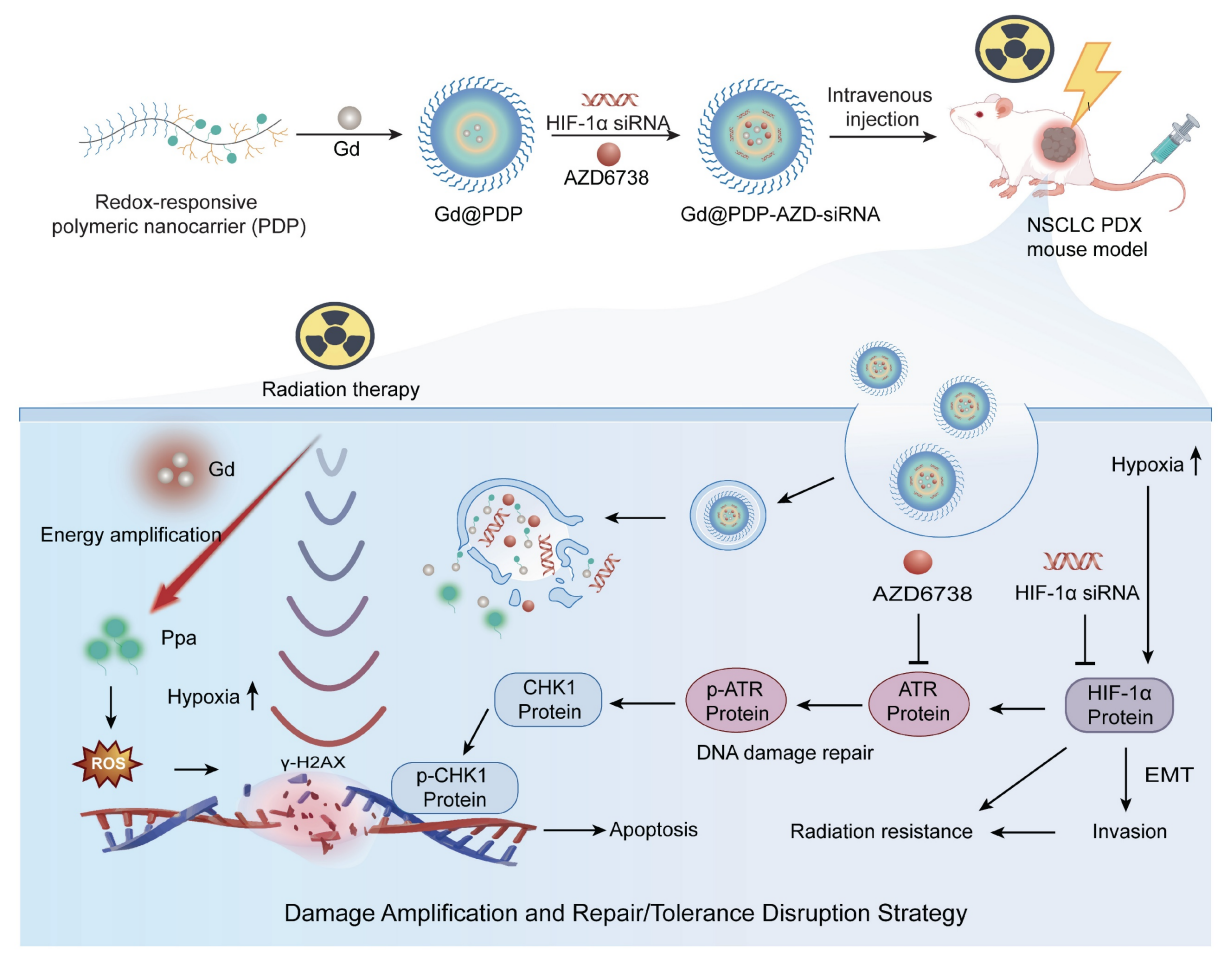
** Design and mechanistic illustration of a Gd-enabled nanoplatform that integrates the amplification of radiodynamic damage with co-inhibition of hypoxia adaptation and ATR checkpoint for radiosensitization of lung adenocarcinoma.** In hypoxic tumors, stabilization of HIF-1α and activation of ataxia-telangiectasia and Rad3-related (ATR)-mediated checkpoint signaling synergistically contribute to adaptive radioresistance. The engineered redox-responsive polymeric nanoplatform enables co-delivery of HIF-1α siRNA and AZD6738, an ATR inhibitor, and realization of a Gd-Ppa radiodynamic modality. Upon X-ray irradiation, Gd³⁺ enhances local energy deposition and facilitates secondary electron generation, thereby activating coordinated Ppa moieties to amplify radiodynamic ROS production and strengthen initial DNA damage. Concurrent suppression of HIF-1α-driven hypoxia adaptation and ATR-dependent checkpoint signaling impairs DNA repair and mitigates damage tolerance. This coordinated intervention approach shifts radiation-induced lesions toward persistent DNA damage and apoptosis, ultimately enhancing radiosensitivity and tumor suppression.

## Abbreviations

RT: radiotherapy
LUAD: lung adenocarcinoma
DDR: DNA damage response
ATR: ataxia-telangiectasia and Rad3-related
Gd: gadolinium
Ppa: pyropheophorbide a
RDT: radiodynamic therapy
ROS: reactive oxygen species
DSBs: DNA double-strand breaks
PDX: patient-derived xenograft
Chk1: Checkpoint kinase 1
high-Z: high atomic number
TCGA: The Cancer Genome Atlas
RAFT: reversible addition-fragmentation chain-transfer
GSH: glutathione
c-PARP: cleaved PARP
SPF: specific pathogen-free
ROI: region-of-interest
