# Supplementary material for: Synergistic nanomedicine overcomes hypoxia-driven DNA repair to potentiate radiotherapy for lung adenocarcinoma
Source: Theranostics. 2026 Mar 17;16(10):5518–36. doi: 10.7150/thno.132916 (PMC13080800; doi:10.7150/thno.132916)
Supplement: Supplementary file 1 — Supplementary materials, instrumentations, methods, figures and tables. [file thnov16p5518s1.pdf]

Supplementary Materials

Synergistic nanomedicine overcomes hypoxia-driven DNA repair to potentiate radiotherapy for lung adenocarcinoma

Zhichang Liu[1#], Yue Qiu[1#], Jie Chen[1#], Jue Li[1], Cheng Zhuang[1], Qiyi Feng[1], Jinxing Huang[1], Chunxiu Xiao[1], Xiuli Zheng[1], Kui Luo[1, 2, 3*], Kai Xiao[1,2,*]

1. Laboratory of Precision Therapeutics, Department of Pulmonary and Critical Care Medicine, State Key Laboratory of Respiratory Health and Multimorbidity, Institution of Radiology and Medical Imaging, Huaxi MR Research Center (HMRRC), Department of Radiology, National Clinical Research Center for Geriatrics, State Key Laboratory of Biotherapy, Frontiers Science Center for Disease-Related Molecular Network, West China Hospital, Sichuan University, Chengdu, 610041, China.
2. Frontier Medical Center, Tianfu Jincheng Laboratory, Chengdu, 610041, China.
3.Psychoradiology Key Laboratory of Sichuan Province, and Research Unit of Psychoradiology, Chinese Academy of Medical Sciences, Chengdu, 610041, China.

[#] These authors contributed equally: Zhichang Liu, Yue Qiu and Jie Chen.
*Corresponding authors: Prof. Kui Luo, E-mail: luokui@scu.edu.cn; Prof. Kai Xiao, E-mail: xiaokaikaixiao@scu.edu.cn.

Contents

1. Materials and instrumentations 3

1.1 Materials 3

1.2 Instrumentations 3

2. Methods 3

2.1 Synthesis and characterization of redox-responsive polymers 3

2.1.1 Synthesis of functional monomers 4

2.1.2 Synthesis of redox-responsive polymers 6

2.2 Preparation and characterization of redox-responsive polymeric nanomedicines 7

2.2.1 Determination of the Ppa content in PDP 7

2.2.2 Preparation of Gd@PDP 8

2.2.3 Preparation of Gd@PDP-AZD 8

2.2.4 Preparation of Gd@PDP-AZD-siRNA 8

2.2.5 Physicochemical characterization of NPs 9

2.2.6 Radiodynamic activity of NPs 9

2.3 *In vitro* cellular assays and radiobiological evaluations 9

2.3.1 Western blot analysis 9

2.3.2 Apoptosis assay 9

2.3.3 Intracellular ROS detection 10

2.3.4 Immunofluorescence 10

2.3.5 Cell invasion assay 10

2.3.6 Colony formation assay 10

3. Supplementary figures 11

4. Supplementary tables 25

1. Materials and instrumentations

1.1 Materials

Unless otherwise stated, routine chemicals and solvents were purchased from commercial suppliers and used as received. CTA, VA-044, and OEGMA were obtained from Sigma-Aldrich (USA). AZD6738 was purchased from APExBIO (USA). Antibodies against HIF-1α and γ-H2AX were obtained from Cell Signaling Technology (USA) and ABclonal (China), respectively. LysoTracker™ Green DND-26 and Hoechst 33342 Ready Flow™ reagent were purchased from Thermo Fisher Scientific (USA). DAPI, DCFH-DA, and crystal violet staining solution were obtained from Beyotime (Shanghai, China). FAM-labeled siRNA, HIF-1α siRNA, and NC siRNA were synthesized by GenePharma (Shanghai, China).

1.2 Instrumentations

Structural and physicochemical characterization was performed using multiple analytical instruments. ^{1}H nuclear magnetic resonance (^{1}H NMR) spectra were obtained on a Bruker 400 MHz spectrometer. High-resolution mass spectrometry (HRMS), liquid chromatography–mass spectrometry (LC–MS), and gel permeation chromatography (GPC) were used for molecular characterization. Ultraviolet–visible (UV–Vis) absorption spectra were collected with a PerkinElmer spectrophotometer. Hydrodynamic size and zeta potential were measured on a Malvern Panalytical Zetasizer Pro.

2. Methods

2.1 Synthesis and characterization of redox-responsive polymers

The synthesis experiments were conducted based on the previously designed synthetic route and experimental protocols [1-3]. The synthesized functional monomer compounds were purified and characterized for their structural confirmation and purity determination via their ^{1}H NMR, LC-MS, and HR-MS **(Figures S1-S10)**.

To synthesize redox-responsive polymers, polyOEGMA-CTA was first synthesized via reversible addition-fragmentation chain transfer (RAFT) polymerization. The characteristic peak of the dithiobenzoic acid group was observed in the aromatic region (**Figure S11**). PolyOEGMA-CTA was used as a chain transfer agent, and a block copolymer, polyOEGMA-block-polyTPEP, was synthesized by

RAFT polymerization (**Figure S12**). After breaking the disulfide bond with DTT, polyOEGMA-block-polySH, with exposed thiol groups, was obtained (**Figure S13**). Finally, through a click reaction (Mal-Ppa) and a substitution reaction (Py-SS-Dendron(G2)-Arg), the final product, polyOEGMA-block-poly[Dendron(G2)-Ppa] (PDP), was obtained (**Figure S14**).

2.1.1 Synthesis of functional monomers

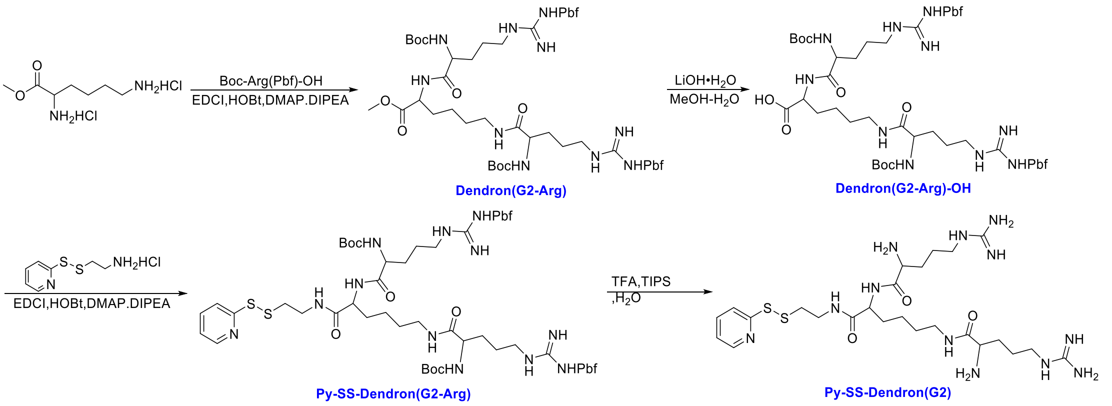

Scheme S1. Preparation of functional and redox-responsive dendron Py-SS-Dendron(G2).

Synthesis of Dendron(G2-Arg). Boc-Arg(Pbf)-OH (7.98 g, 15.15 mmol), EDCI (5.90 g, 30.78 mmol), HOBt (4.11 g, 30.42 mmol), and DMAP (1.89 g, 15.47 mmol) were dissolved in anhydrous DCM (150 mL). The mixture was stirred in an ice bath for 15 min, then DIPEA (10.80 mL, 60.47 mmol) and L-lysine methyl ester dihydrochloride (1.55 g, 6.65 mmol) were added under mildly basic conditions. After stirring for 24 h, the mixture was worked up, and dried over Na_2SO_4, and concentrated. A crude product was purified to afford a whitish solid (ca. 7.00 g, yield: 89.40%).

^{1}H NMR (400 MHz, ***d6***-DMSO) δ 8.15 (d, *J* = 7.3 Hz, 1H), 7.78 (t, *J* = 5.5 Hz, 1H), 6.82 (d, *J* = 8.1 Hz, 2H), 6.76 (d, *J* = 8.1 Hz, 1H), 6.65 (s, 2H), 6.38 (s, 3H), 4.19 (dd, *J* = 13.3, 8.1 Hz, 1H), 3.98 – 3.87 (m, 1H), 3.82 (dd, *J* = 13.2, 7.9 Hz, 1H), 3.59 (s, 3H), 3.01 (s, 6H), 2.96 (s, 4H), 2.47 (s, 6H), 2.42 (s, 6H), 2.00 (s, 6H), 1.75 – 1.63 (m, 1H), 1.62 – 1.49 (m, 3H), 1.38 (d, *J* = 16.2 Hz, 40H). (**Figure S1**). LC-MS (ES+): m/z = 589.3 $[M/2+H]^+$, m/z = 1177.2 $[M+H]^+$ (**Figure S2**). HR-MS: calcd for $C_{55}H_{88}N_{10}O_{14}S_2$ $[M/2+2H]^{2+}$ 590.3118, found 590.3074 (**Figure S3**).

Synthesis of Dendron(G2-Arg)-OH. Dendron (G2-Arg) (5.38 g, 4.57 mmol) was

dissolved in methanol/water, and $LiOH \cdot H_2O$ (0.97 g, 23.12 mmol) was added. The mixture was stirred overnight until complete reaction. After evaporation, the residue was dissolved in DCM, acidified with dilute HCl, and extracted. The organic phase was washed with brine, dried over Na_2SO_4, and concentrated to afford a white solid (5.28 g, yield: 99.25%).

^{1}H NMR (400 MHz, ***d6***-DMSO) δ 12.53 (s, 1H), 7.98 (d, *J* = 7.4 Hz, 1H), 7.82 (t, *J* = 5.3 Hz, 1H), 6.84 (d, *J* = 8.1 Hz, 4H), 6.76 (d, *J* = 8.2 Hz, 1H), 6.43 (s, 3H), 4.17 – 4.13 (m, 1H), 3.97 – 3.87 (m, 1H), 3.82 (dd, *J* = 13.2, 7.9 Hz, 1H), 3.01 (d, *J* = 5.7 Hz, 6H), 2.96 (s, 4H), 2.47 (s, 6H), 2.42 (s, 6H), 2.00 (s, 6H), 1.69 (dt, *J* = 19.7, 5.7 Hz, 1H), 1.56 (ddd, *J* = 19.7, 10.1, 6.1 Hz, 3H), 1.48 – 1.25 (m, 40H).(**Figure S4**). LC-MS (ES+): m/z = 582.2 $[M/2+H]^+$ (**Figure S5**). HR-MS: calcd for $C_{54}H_{86}N_{10}O_{14}S_2$ $[M/2+2H]^{2+}$ 583.3039, found 583.2964 (**Figure S6**).

Synthesis of Py-SS-Dendron (G2-Arg). Dendron (G2-Arg)-OH (5.28 g, 4.54 mmol), EDCI (1.86 g, 9.55 mmol), HOBt (1.24 g, 9.18 mmol), and DMAP (0.56 g, 4.58 mmol) were dissolved in approximately 200 mL anhydrous DCM. DIPEA (4.00 mL, 2.92 g, 22.59 mmol) and Py-SS-$NH_2 \cdot$HCl (1.55 g, 6.96 mmol) were added, under weakly basic condition. After stirring for 24 h, the mixture was worked up, purified by column chromatography, and afforded a white solid (ca. 3.90 g, yield: 64.57%).

^{1}H NMR (400 MHz, ***d6***-DMSO) δ 8.45 (d, *J* = 4.7 Hz, 1H), 8.14 (s, 1H), 8.04 (s, 1H), 7.86 – 7.70 (m, 4H), 7.29 – 7.17 (m, 1H), 7.02 (d, *J* = 6.7 Hz, 1H), 6.93 (d, *J* = 7.9 Hz, 1H), 6.73 (d, *J* = 8.1 Hz, 1H), 6.65 (s, 1H), 6.37 (s, 3H), 4.17 (d, *J* = 6.3 Hz, 1H), 3.92 – 3.73 (m, 2H), 2.97 (d, *J* = 16.6 Hz, 10H), 2.88 (dd, *J* = 13.0, 6.7 Hz, 2H), 2.47 (s, 6H), 2.42 (s, 6H), 2.00 (s, 6H), 1.66 – 1.06 (m, 46H) (**Figure S7**). LC-MS (ES+): m/z = 1331.4 $[M+H]^+$ (**Figure S8**). HR-MS: calcd for $C_{61}H_{94}N_{12}O_{13}S_4$ $[M+H]^+$ 1331.6024, found 1331.6020 (**Figure S9**).

Synthesis of Py-SS-Dendron(G2). Py-SS-Dendron(G2-Arg) (1.50 g, 1.13 mmol) was placed in an ice-water bath, followed by the addition of TFA (14.25 mL), and the formulation was agitated until fully dissolved. After Py-SS-Dendron was completely dissolved, TIPS (375 μL) and water (H_2O, 375 μL) were added. After 30 min, the formulation was agitated for one days. The product was quenched, and the solvent was removed. The residue was washed with DCM three times, then added dropwise into ice-cold diethyl ether to precipitate a white solid. The solid was collected by

centrifugation and dried under vacuum (1.03 g, yield 84.43%). HR-MS: calcd for $C_{25}H_{46}N_{12}O_3S_2$ $[M+H]^+$ 627.3335, found 627.3332 (**Figure S10**).

2.1.2 Synthesis of redox-responsive polymers

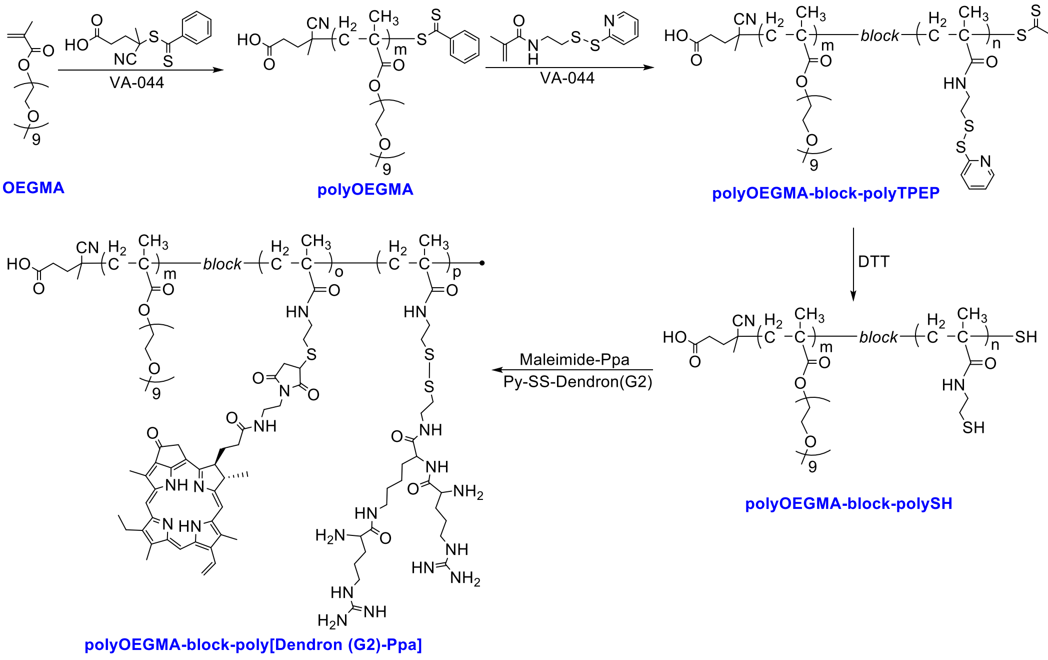

Scheme S2. Synthesis routes of PDP.

Synthesis of polyOEGMA. OEGMA (5.02 g), CTA (38 mg) and VA-044 (19 mg) were dissolved in a solvent mixture (methanol/water = 5:1). The solution was sealed, ice cooled, stirred, and purged with nitrogen for 30 min. The mixture was then stirred in the dark at 47 °C for 20 h before quenching with liquid nitrogen. The reaction product was dialyzed (2 kDa MWCO) at 4 °C for two days to remove unreacted starting materials. The dialysate was freeze-dried under vacuum to yield approximately 4.70 g of a pink oily liquid with a yield of 92.92%. The molecular weight of the product was characterized by GPC, with Mn = 22.6 kDa, Mw = 28.9 kDa, and PDI = 1.28. Its NMR spectrum is shown in **Figure S11**.

Synthesis of polyOEGMA-block-polyTPEP. PolyOEGMA (3.03 g, 0.1 mmol), TPEP (1.06 g, 4.18 mmol), and VA-044 (14 mg, 0.04 mmol) were solubilized in 9 mL methanol and of 1 mL water. The solution was sealed, ice-cooled, and purged with nitrogen for 40 min. The combination was then transferred to a 47 °C oil bath and stirred in the dark for 24 h. The combination reaction was quenching with liquid nitrogen. The product was dialyzed (2 kDa MWCO) at 4 °C for 48 h, filtered (φ = 0.45 μm) and vacuum freeze-dired to afford 3.85 g of a pink oily liquid (yield: 94.13%). The

molecular weight of the product was characterized by GPC, with Mn = 28.3 kDa, Mw = 36.0 kDa, and PDI = 1.27. Its NMR spectrum is shown in **Figure S12**.

Synthesis of polyOEGMA-block-polySH. PolyOEGMA-block-polyTPEP (2.00 g) was dispersed in 20 mL of RO water in a round-bottom flask under stirring. After adding DTT (1.50 g), the flask was capped and stirred. The next day, the reaction was terminated, and the solution was dialyzed (2 kDa MWCO) at 4 °C for two days. The dialysate was filtered (φ = 0.45 μm) and freeze-dried under vacuum to yield approximately 1.65 g of an oily liquid with a yield of 94.29%. The molecular weight of the product was characterized by GPC, with Mn = 22.5 kDa, Mw = 24.7 kDa, and PDI = 1.10. Its NMR spectrum is shown in **Figure S13**.

Synthesis of polyOEGMA-block-poly[Dendron(G2)-Ppa]. PolyOEGMA-block-polySH (1.67 g) was charged in a round-bottom flask, and DMSO (30 mL) was added. The flask was sealed and subjected to five vacuum–nitrogen purge cycles. Mal-Ppa (130 mg) and a catalytic amount of triethylamine were added to ensure a weakly basic solvent system. The reaction was stirred overnight. Acetic acid was added to adjust the solution to be weakly acidic, and Py-SS-Dendron(G2)-Arg (1.70 g) was added. The reaction was quenched after stirring for 36 h. The solution was dialyzed (2 kDa MWCO) at 4 °C for two days. The dialysate was filtered (φ = 0.45 μm) and freeze-dried under vacuum to yield approximately 2.40 g of a dark green viscous liquid. The molecular weight of the product was characterized by GPC, with Mn = 24.7 kDa, Mw = 28.8 kDa, and PDI = 1.17. Its NMR spectrum is shown in **Figure S14**.

2.2 Preparation and characterization of redox-responsive polymeric nanomedicines

2.2.1 Determination of the Ppa content in PDP

Preparation of a Ppa standard curve. The Ppa solution was serially diluted to prepare solutions at 12.50, 6.25, 3.125, 1.56, and 0.78 μg/mL. The absorption spectra were measured on a UV-Vis spectrophotometer. The maximum absorption wavelength was identified, and the corresponding absorption values were recorded.

Determination of the Ppa content in PDP. A certain amount of PDP was weighed and dissolved in DMSO. The absorbance of the PDP solution at the maximum absorption wavelength determined from the standard curve was measured and recorded.

The Ppa content was calculated by fitting the absorbance value into the standard curve.

2.2.2 Preparation of Gd@PDP

Preparation of Gd@PDP. A predetermined amount of PDP was weighed and dissolved in RO water with stirring. A predetermined amount of gadolinium chloride hexahydrate solution was added, and stirred overnight. Finally, the reaction solution was dialyzed at 4 °C for 48 h. The synthesis route and chemical structure of Gd@PDP are shown in **Scheme S3**. The Gd content was determined by Inductively Coupled Plasma Mass Spectrometry (ICP-MS).

Determination of the Gd Content in Gd@PDP. The Gd content was calculated to be 0.019 mg/L per 100 mg/L Gd@PDP sample, corresponding to a weight percentage (W%) of 0.019%.

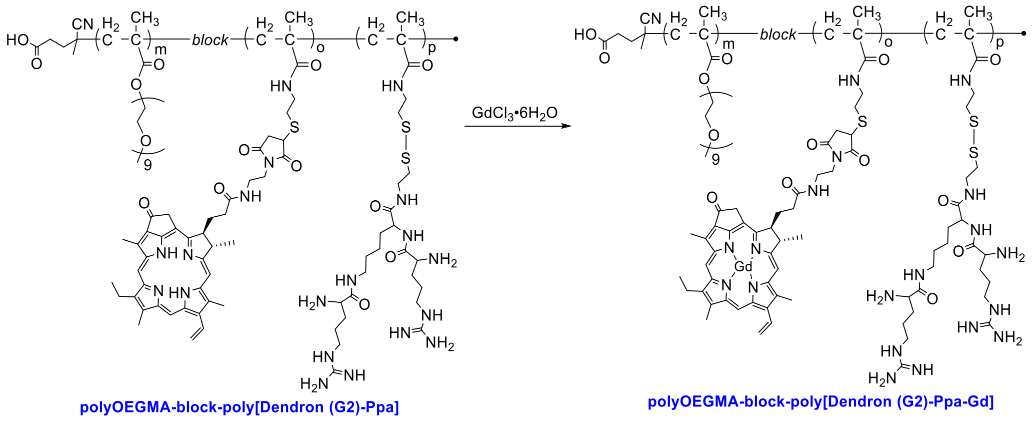

Scheme S3. Synthetic routes and chemical structures of Gd@PDP.

2.2.3 Preparation of Gd@PDP-AZD

Preparation of Gd@PDP-AZD. Gd@PDP-AZD was prepared from Gd@PDP via a thin-film hydration method. A certain amount of AZD6738 dissolved in chloroform was added to the reaction flask. Chloroform was concentrated by rotary evaporation. Finally, RO water was added for sonication. The drug loading content (LC) and the encapsulation efficiency (EE) of AZD6738 in Gd@PDP-AZD nanoparticles were determined by HPLC.

2.2.4 Preparation of Gd@PDP-AZD-siRNA

Preparation of Gd@PDP-AZD-siRNA. Gd@PDP-AZD-siRNA NPs were prepared via electrostatic complexation. A stock solution of siRNA (20 μM in Enzyme-

free water) was added dropwise to Gd@PDP-AZD NPs under vortexing and incubated at room temperature for 40 min to form complexes. The mass ratio of carrier-to-siRNA was varied from 25:1 to 400:1. For fluorescence imaging experiments, FAM-labeled siRNA was used to replace siRNA in the above protocol at a carrier-to-siRNA ratio of 200:1. siRNA complexation and stability were assessed by agarose gel (2%) retardation assays. Reduction-responsive siRNA release was studied by incubating Gd@PDP-AZD-siRNA with DTT (0-20 mM) for 30 min, followed by gel electrophoresis.

2.2.5 Physicochemical characterization of NPs

Morphological analysis. Aqueous solutions of PDP, Gd@PDP, Gd@PDP-AZD, and Gd@PDP-AZD-siRNA were prepared at 1 mg/mL. The hydrodynamic size and zeta potential of each nanocomposite were measured via the Malvern Zetasizer Pro. The morphology of Gd@PDP and Gd@PDP-AZD-siRNA at the same concentration were observed using TEM.

2.2.6 Radiodynamic activity of NPs

Radiodynamic activity was evaluated via a singlet oxygen sensor, 9,10-anthracenediyl-bis(methylene)dimalonic acid (ABDA). The nanoparticle solution was mixed with ABDA and then irradiated (0–8 Gy). The degradation of ABDA was monitored by measuring absorbance values at 378 nm using a UV-vis spectrophotometer.

2.3 *In vitro* cellular assays and radiobiological evaluations

2.3.1 Western blot analysis

Western blotting was performed as described previously [2]. Equal amounts of protein from each treatment group were subjected to electrophoresis, transferred to a membrane, and blocked with 5% skim milk. The membrane was incubated with primary antibody overnight at 4 °C, followed by incubation with secondary antibody at room temperature the next day. Protein bands were visualized using chemiluminescence detection.

2.3.2 Apoptosis assay

A549 cells were seeded and allowed to adhere overnight. Cells were treated with different nanoparticle formulations for 6 h under hypoxic conditions (1% O_2), followed

by irradiation (4 Gy). After incubation for additional 72 h, cells were harvested and stained with Annexin V-FITC/PI according to the manufacturer's protocol. Apoptotic cells were analyzed using a CytoFLEX flow cytometer.

2.3.3 Intracellular ROS detection

Intracellular ROS levels were measured as described previously [2]. Briefly, cells were incubated with DCFH-DA dye at a specified ratio in culture medium at 37 °C for 25 min. After washing three times with PBS, nuclei were counterstained with Hoechst 33342. Fluorescence images were then acquired.

2.3.4 Immunofluorescence

Immunofluorescence staining was performed as described previously [2]. Following treatment, cells were fixed with 4% paraformaldehyde, permeabilized with Triton X-100, and blocked with BSA. After washing three times with PBS, the cells were incubated with primary antibodies at appropriate dilutions overnight at 4 °C. On the next day, the primary antibodies were removed, and corresponding fluorescent secondary antibodies were added. After incubation, the cells were washed three times with PBS, and the nuclei were stained with DAPI. Fluorescence images were then acquired.

2.3.5 Cell invasion assay

A549 cells were treated with the indicated nanoformulations for 6 hours prior to radiotherapy. After treatment, cells were seeded at a density of 5×10^4 cells per well into the upper chambers of Transwell inserts coated with Matrigel. After another 24-hour incubation, cells that had invaded through the filter membrane were fixed and stained with crystal violet.

2.3.6 Colony formation assay

A549 cells were seeded at 500 cells per well in 6-well plates and treated with different nanoformulations the following day. After 6 hours of drug treatment, cells were exposed to radiotherapy. Cells were then transferred to a hypoxic culture conditions and incubated for 14 days. Finally, colonies were fixed and stained with crystal violet for imaging.

3. Supplementary figures

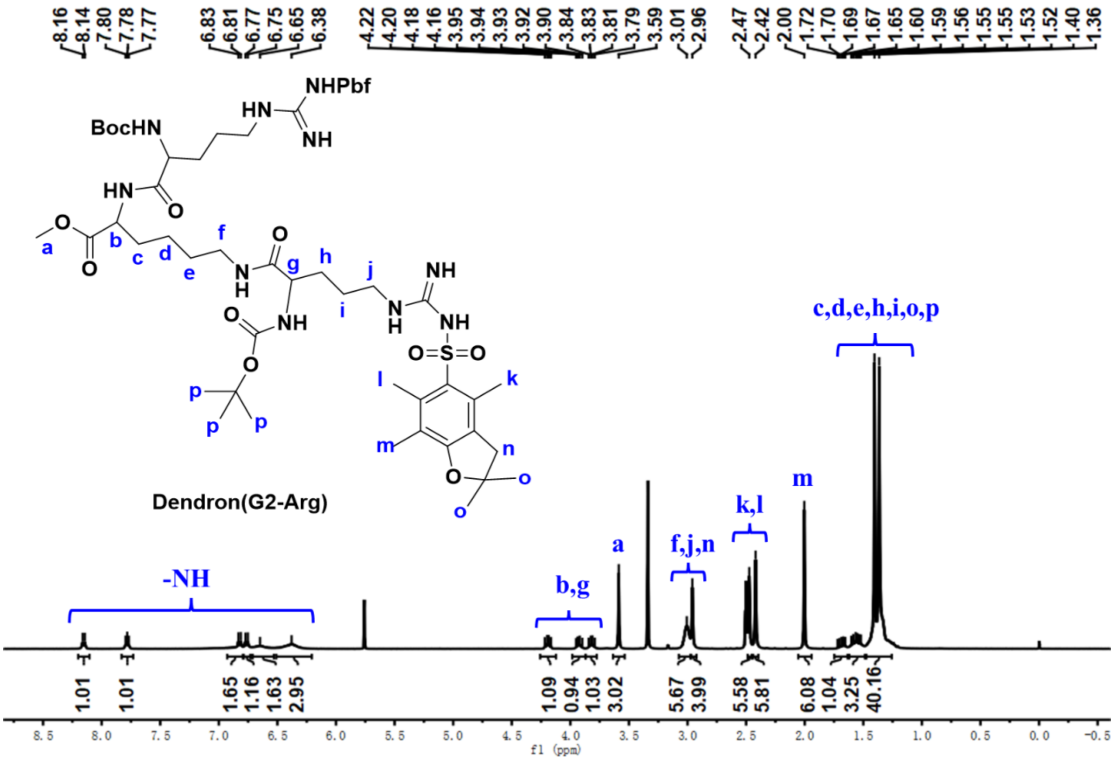

Figure S1. ^{1}H NMR characterization of Dendron (G2-Arg) (recorded in *d6*-DMSO).

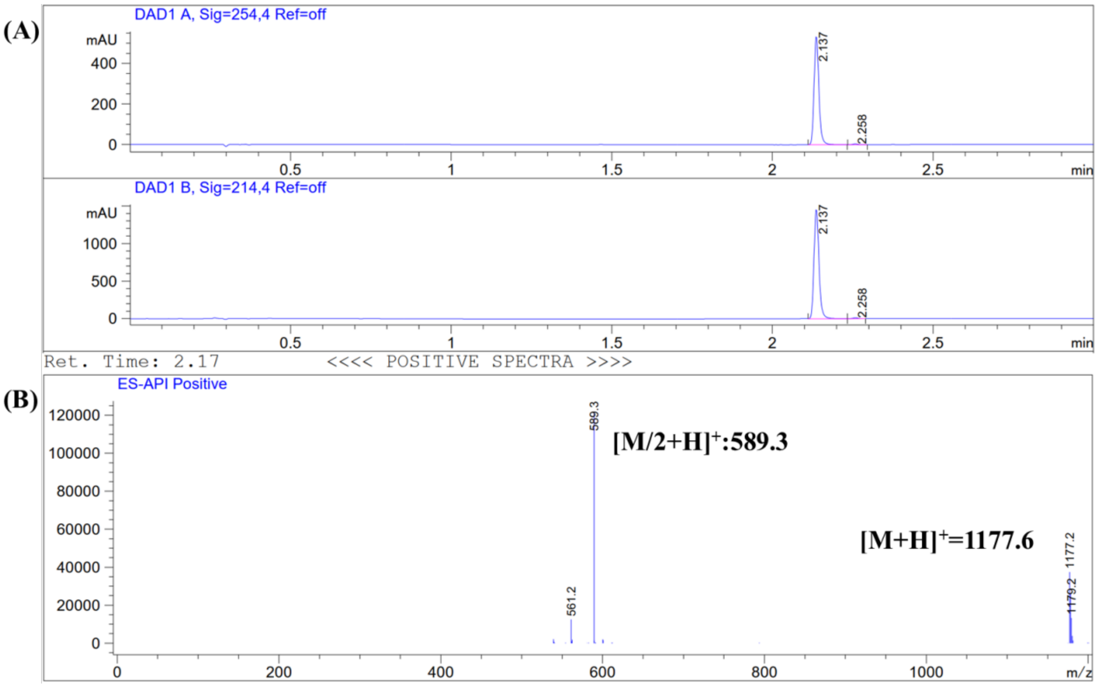

Figure S2. LC-MS spectrum of Dendron (G2-Arg). The product peak at 2.14 min (A), and a single ion peak at 589.3 m/z for $[M/2+H]^+$ and 1177.2 m/z for $[M+H]^+$ (B)

(recorded in an acid).

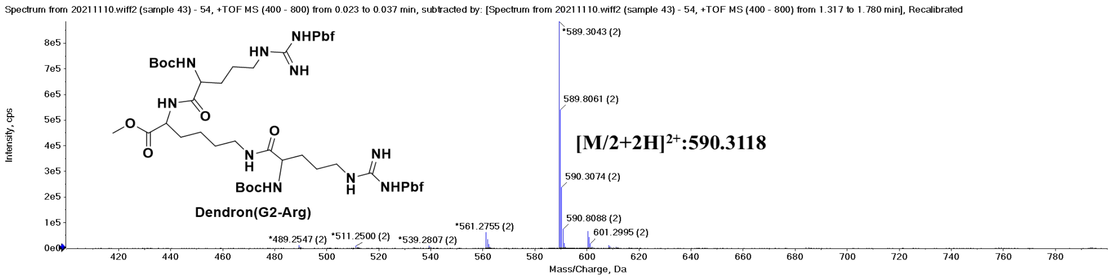

Figure S3. HRMS spectrum of Dendron (G2-Arg). The ion peak at 590.3118 m/z for $[M/2+2H]^{2+}$.

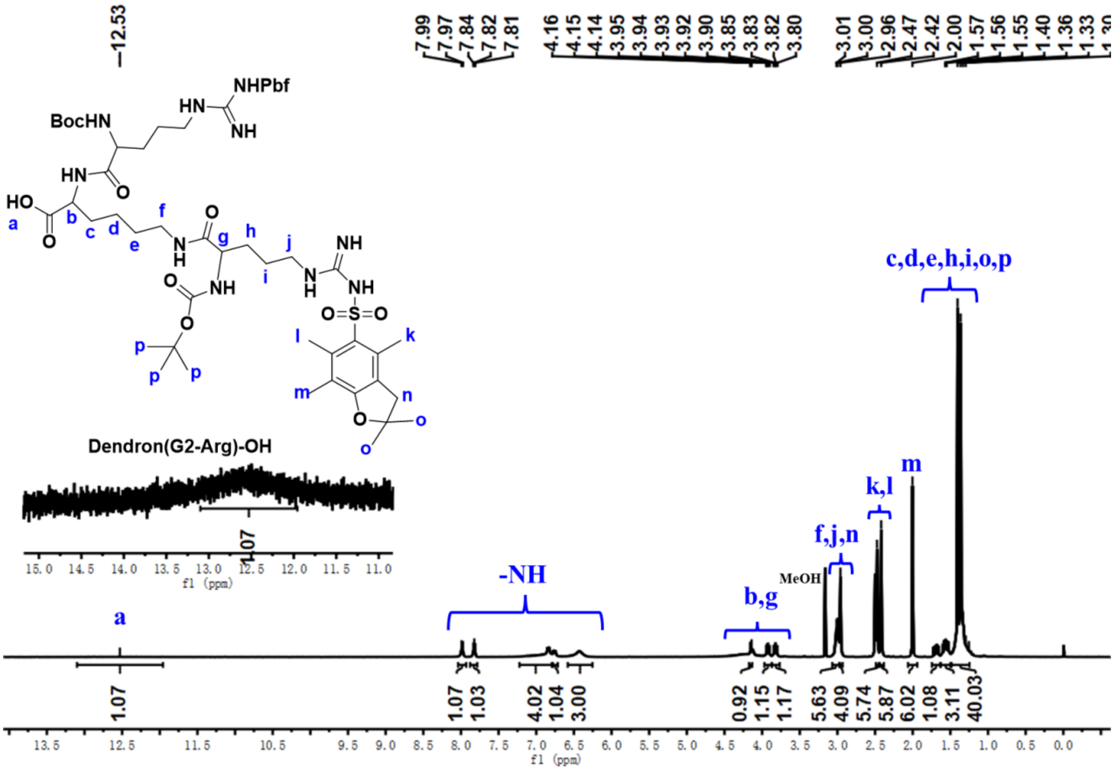

Figure S4. ^{1}H NMR spectrum of Dendron (G2-Arg)-OH (recorded in *d6*-DMSO).

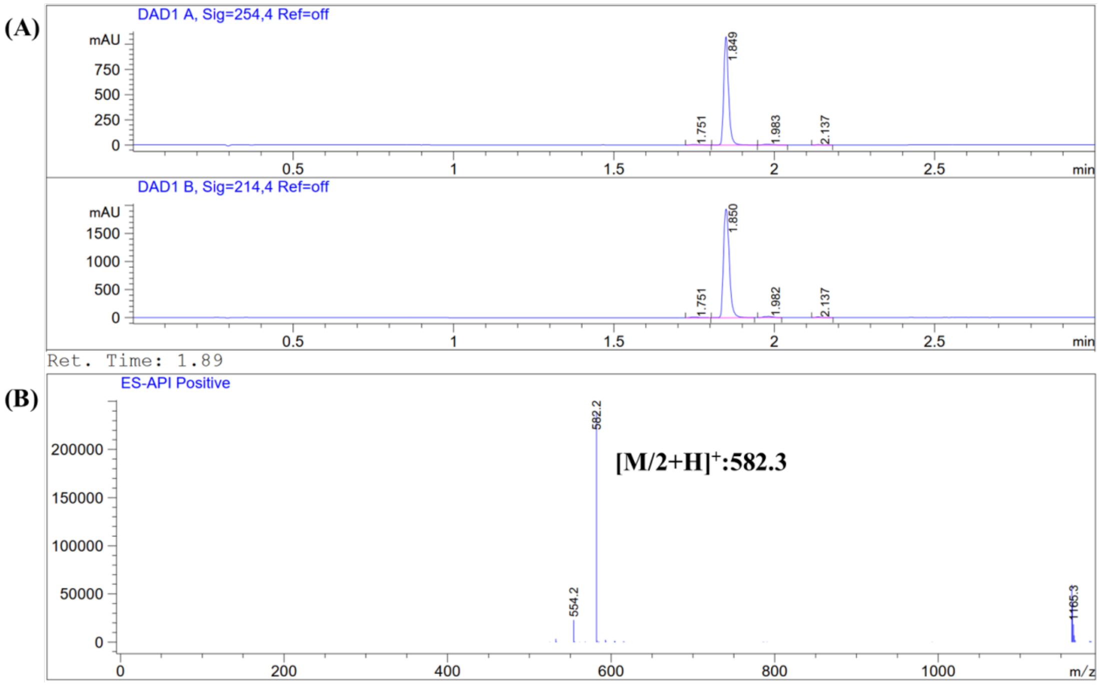

Figure S5. LC-MS spectrum of Dendron (G2-Arg)-OH. The product peak at 1.85 min (A), and a single ion peak at 582.2 m/z for $[M/2+H]^+$ (B) (recorded in an acid).

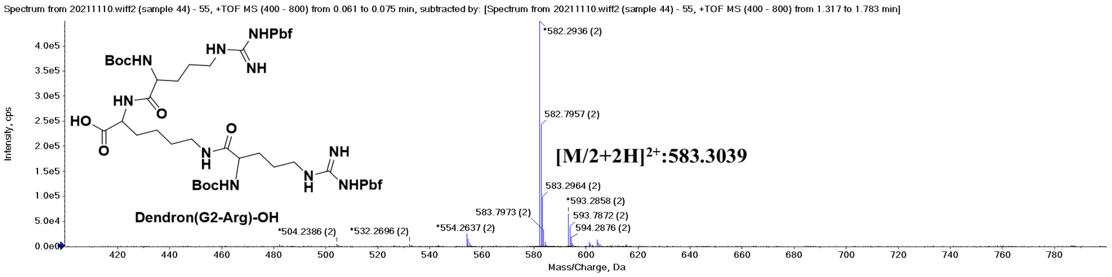

Figure S6. HRMS characterization of Dendron (G2-Arg)-OH. The ion peak at 583.2964 m/z for $[M/2+2H]^{2+}$.

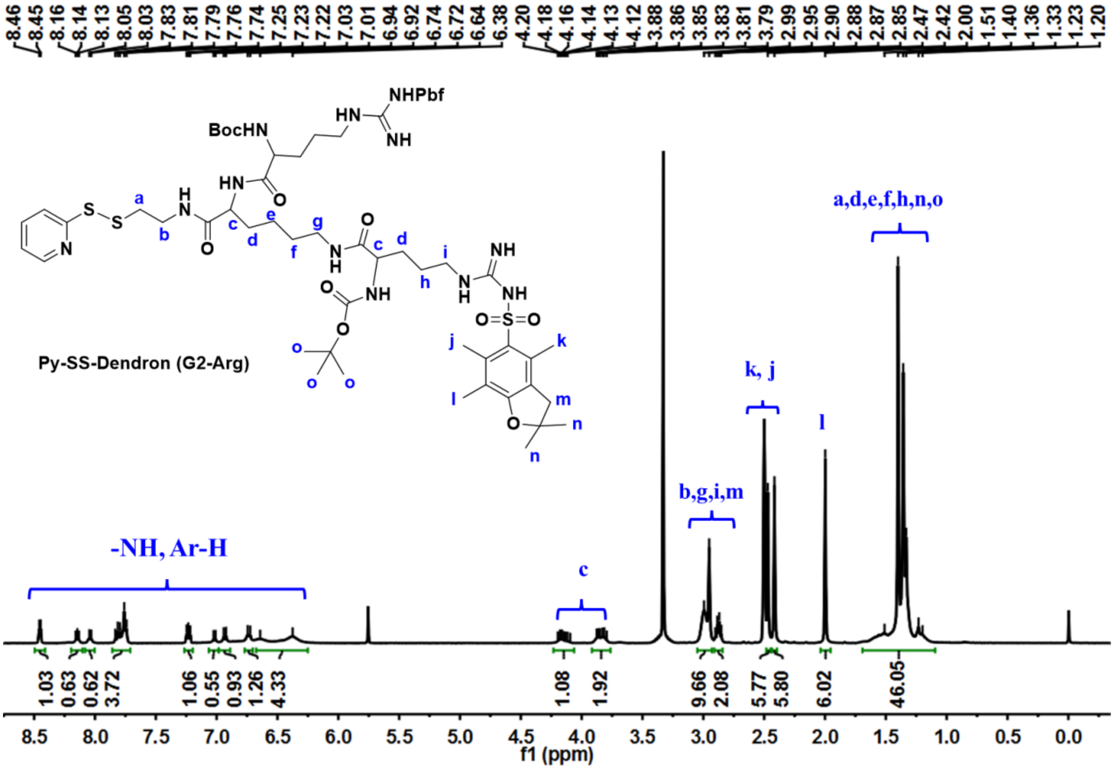

Figure S7. ^{1}H NMR characterization of Py-SS-Dendron (G2-Arg) (recorded in *d6*-DMSO).

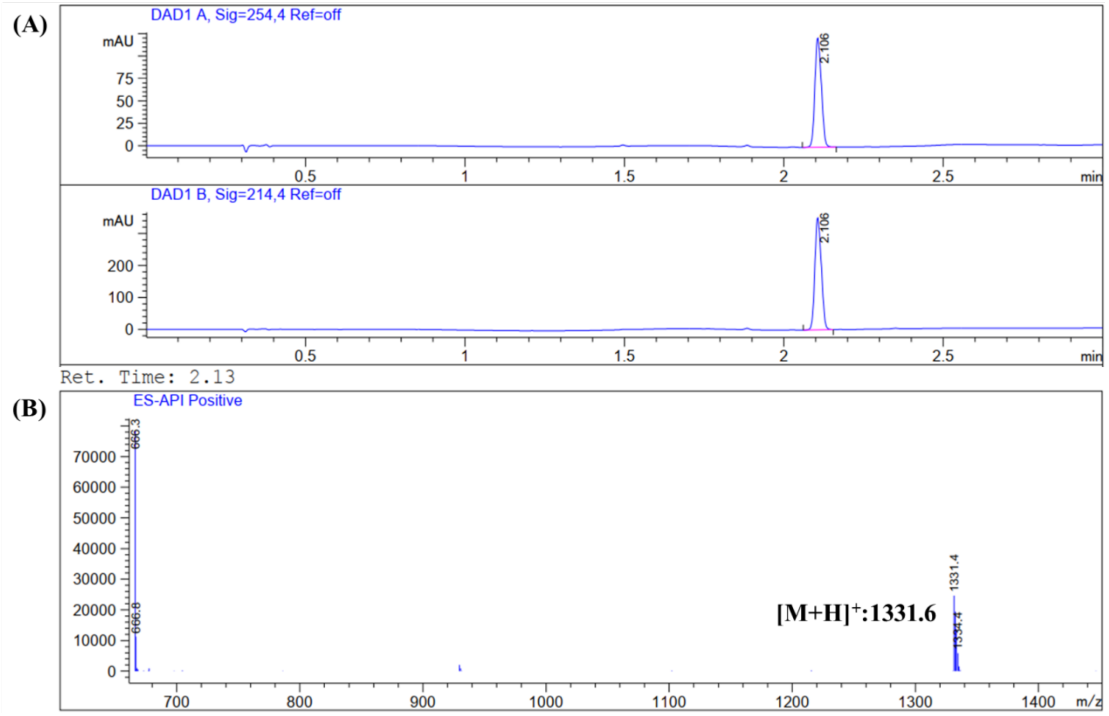

Figure S8. LC-MS characterization of Py-SS-Dendron (G2-Arg). The product peak at 2.11 min (A), and a single ion peak at 1331.4 m/z for $[M+H]^{+}$ (B) (recorded in an acid).

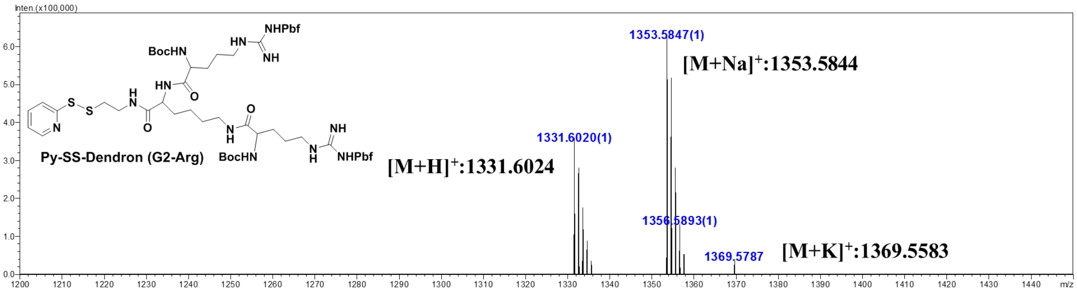

Figure S9. HRMS characterization of Py-SS-Dendron (G2-Arg). The ion peak at 1331.6020 m/z for $[M+H]^+$, 1353.5847 m/z for $[M+Na]^+$ and 1369.5787 m/z for $[M+K]^+$.

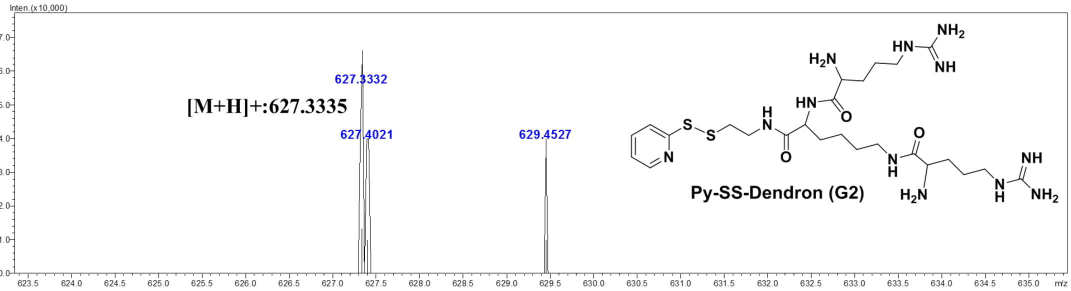

Figure S10. HRMS characterization of Py-SS-Dendron (G2). The ion peak at 627.3332 m/z for $[M+H]^+$.

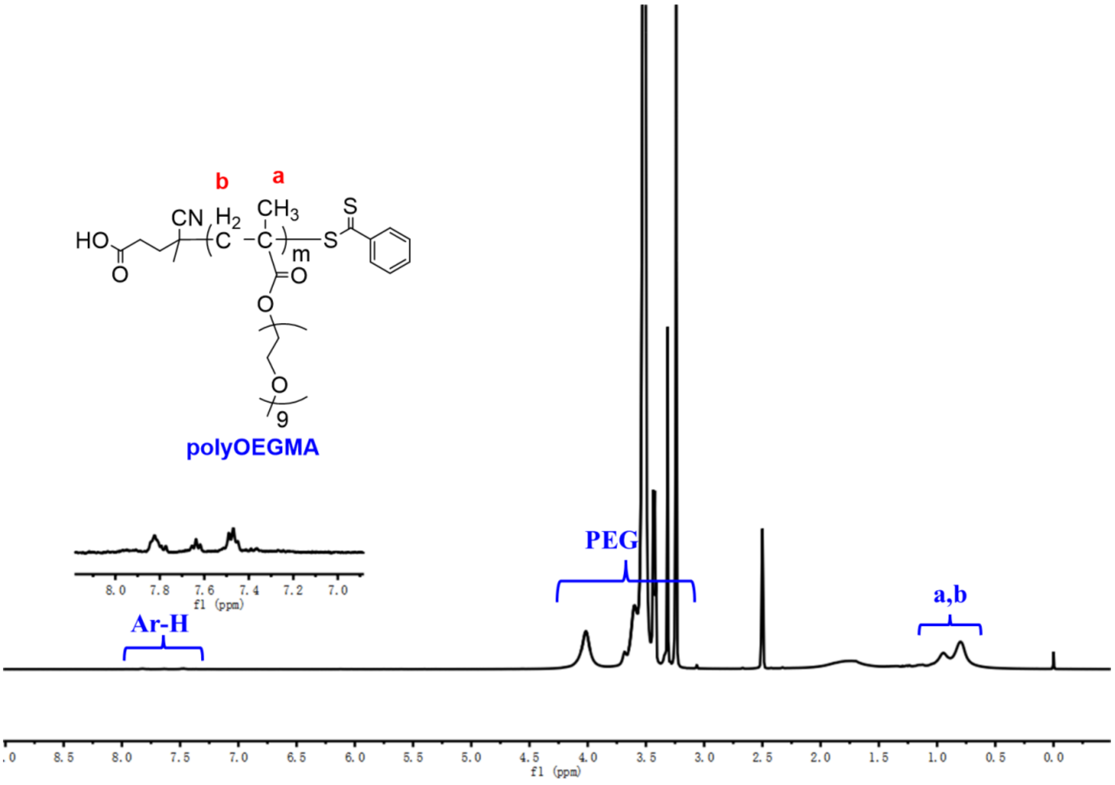

Figure S11. ^{1}H NMR characterization of polyOEGMA (recorded in *d6*-DMSO).

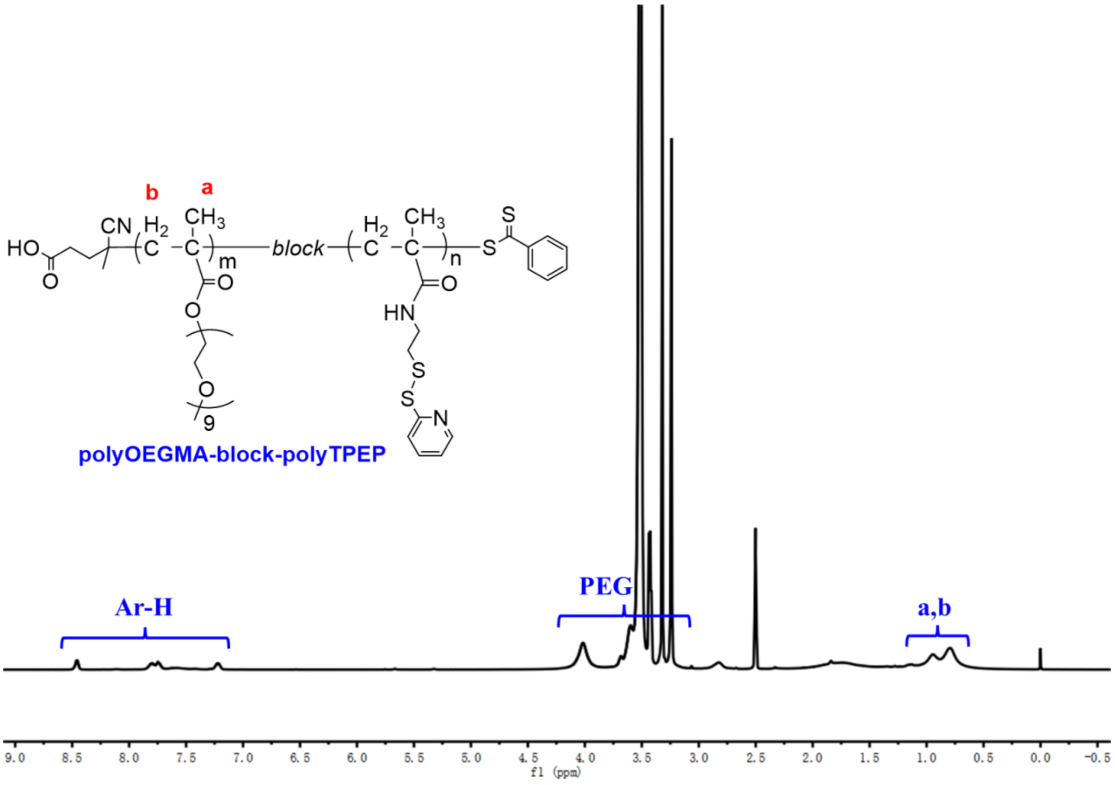

Figure S12. ^{1}H NMR of characterization polyOEGMA-block-polyTPEP (recorded in *d6*-DMSO).

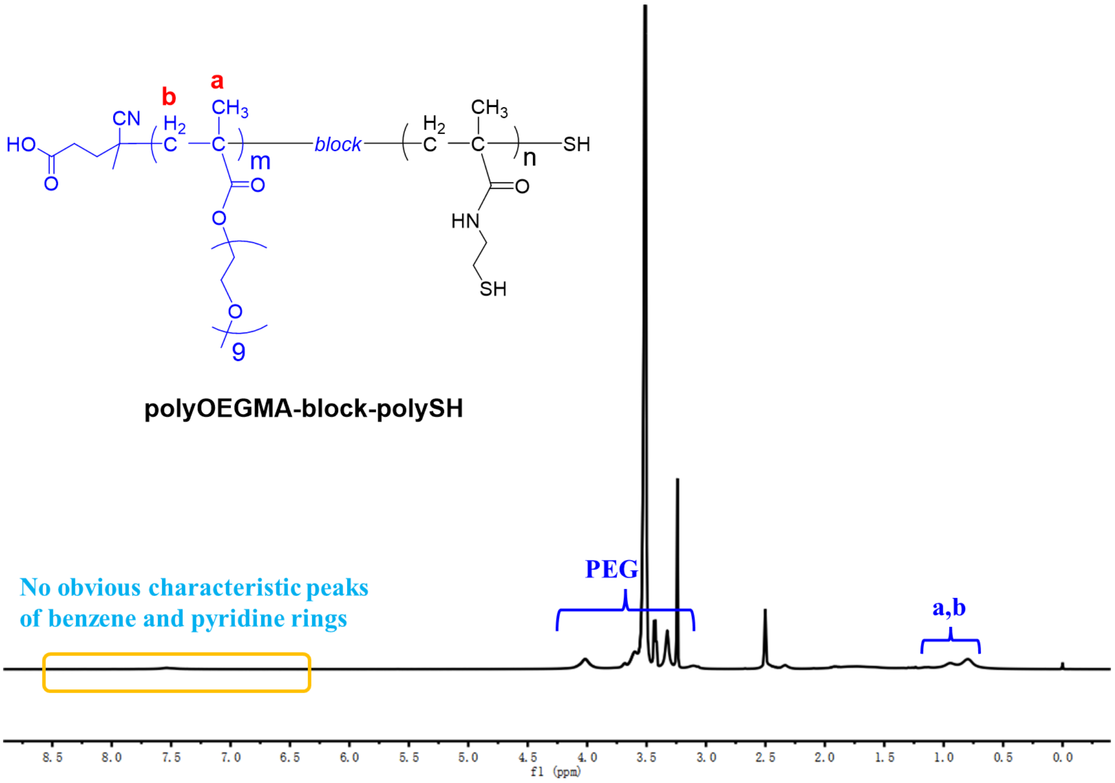

Figure S13. ^{1}H NMR characterization of polyOEGMA-block-polySH (recorded in *d6*-DMSO).

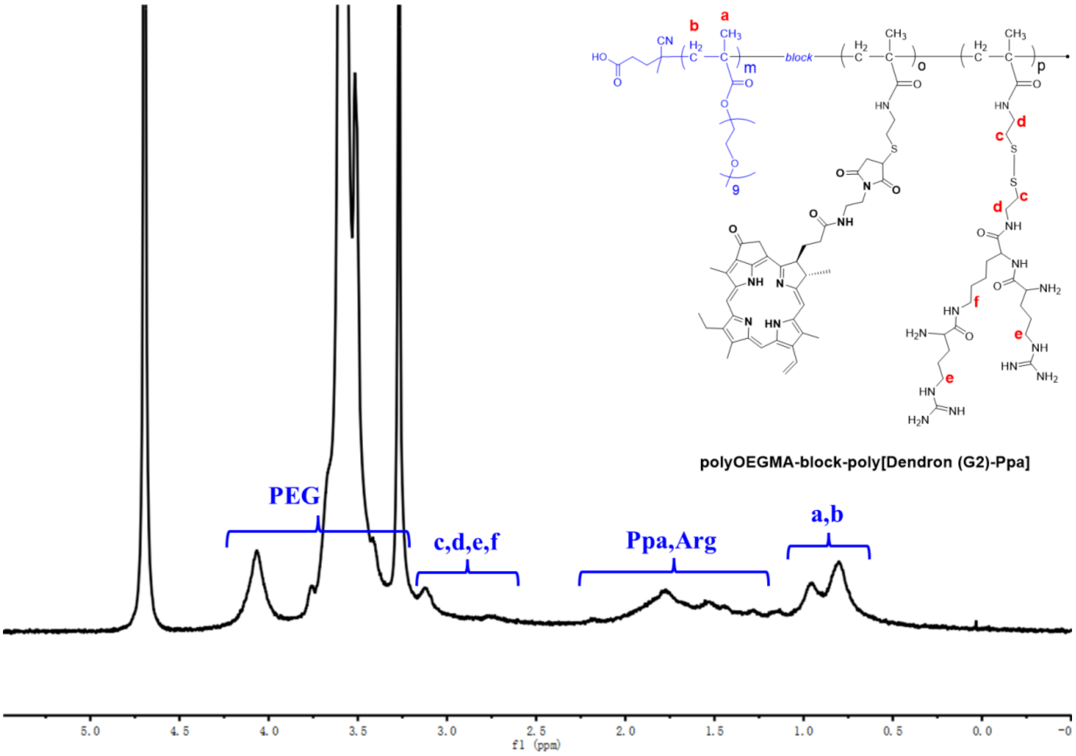

Figure S14. ^{1}H NMR characterization of polyOEGMA-block-poly[Dendron(G2)-Ppa] (recorded in *d6*-DMSO).

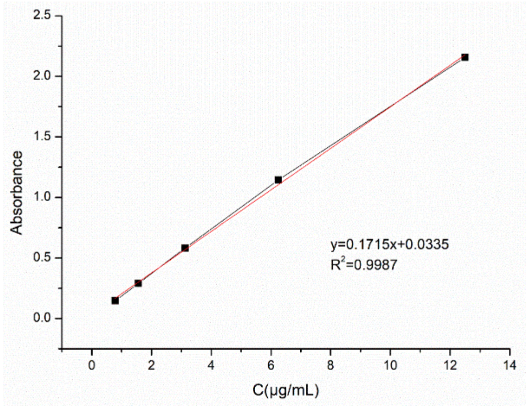

Figure S15. The UV-Vis standard curve of Ppa.

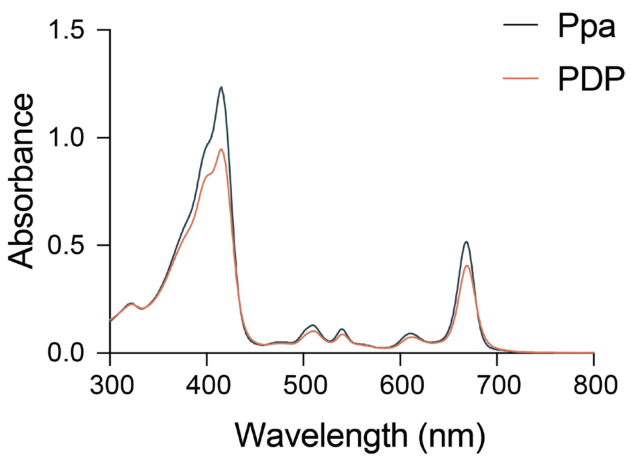

Figure S16. UV–Vis absorption spectra of free Ppa and PDP polymer.

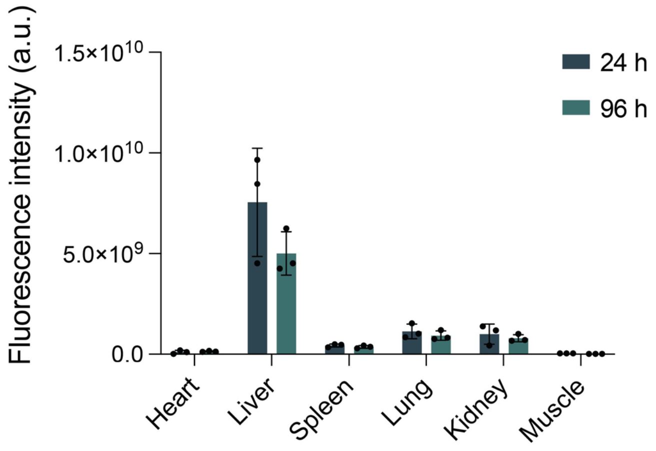

Figure S17. Quantitative biodistribution of PDP in healthy mice. Fluorescence signals in major organs were assessed at 24 and 96 h following intravenous administration (n = 3). Mean ± SD.

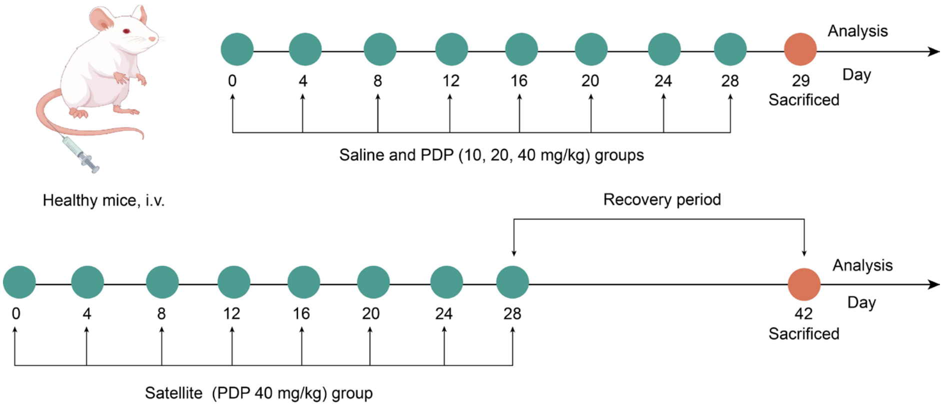

Figure S18. Experimental design for PDP systemic toxicity assessment. Fifty BALB/c mice, equally distributed by sex, were randomly allocated into five experimental groups (10 animals per group). The animals received intravenous injections of either saline or PDP at doses of 10, 20, or 40 mg/kg once every 4 days over a 28-day period. A 40 mg/kg satellite group was included to assess

recovery over a 14-day washout period. Primary cohorts were sacrificed on day 29 for toxicological analysis, while the satellite group was evaluated at the study endpoint (day 42).

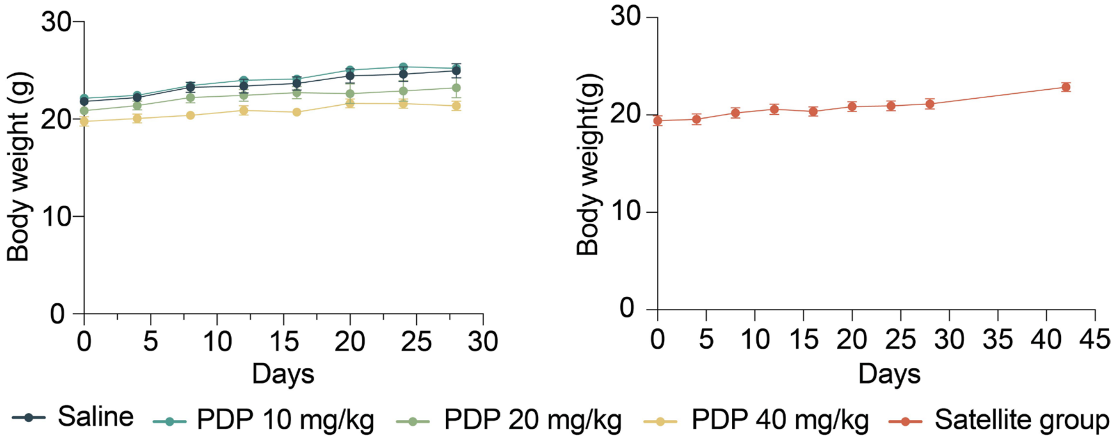

Figure S19. Systemic toxicity assessment of PDP. Body weight changes in mice during (left) the 28-day repeated-dose period (treatment groups) and (right) the subsequent 14-day recovery period (the satellite group) (n = 5). Mean ± SD.

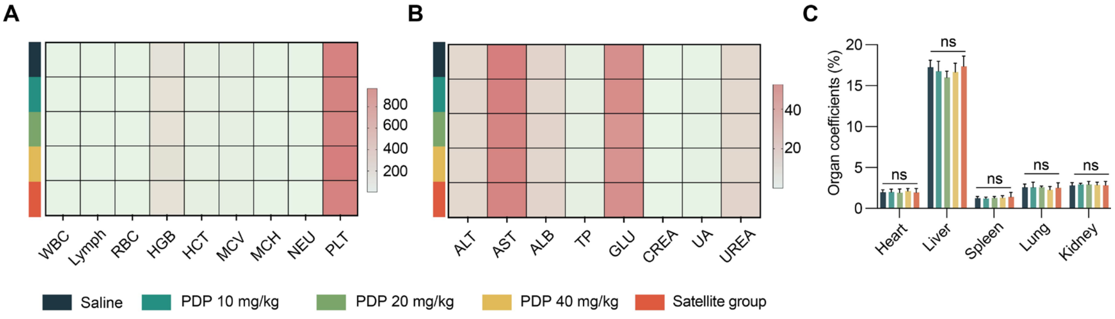

Figure S20. Baseline physiological parameters in tumor-free female mice after PDP administration. (A) Complete blood counts parameters (CBC) (n = 5). (B) Serum biochemical markers for the hepatic and renal function (n = 5). (C) Organ-to-body weight ratios (n = 5). Mean ± SD; ns, not significant.

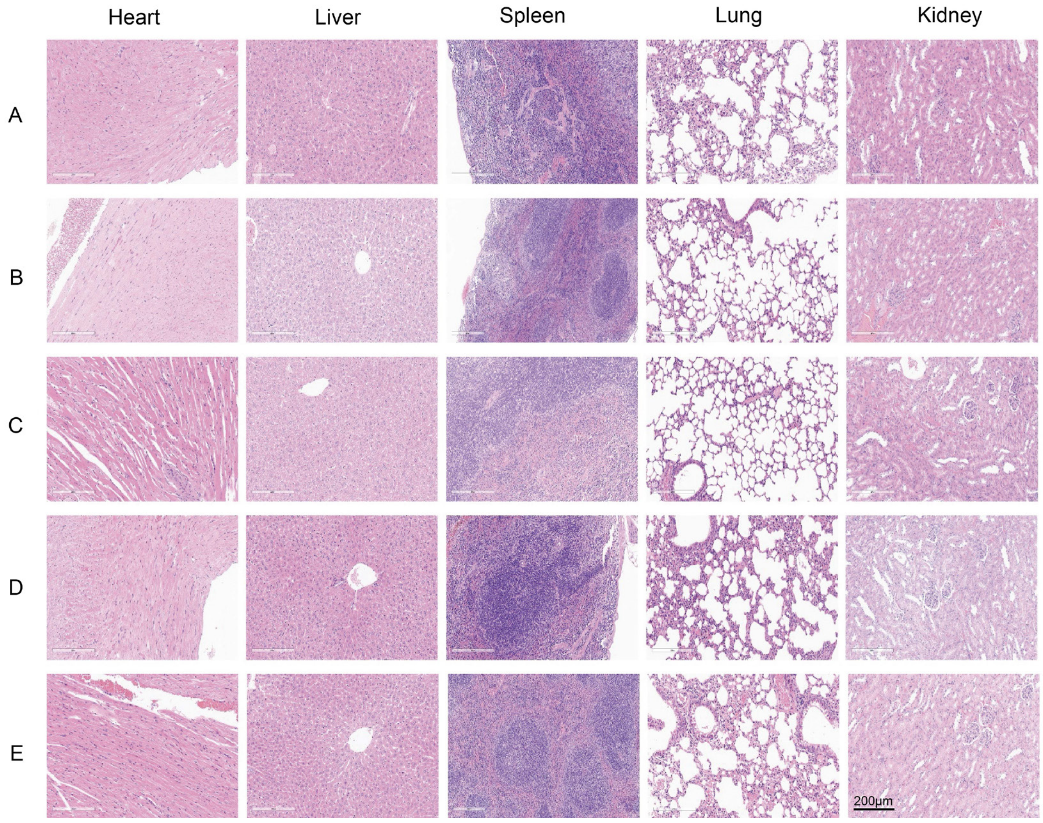

Figure S21. Representative H&E-stained images of the heart, liver, spleen, lung, and kidney from mice in the systemic toxicity study. The top to bottom rows are: (A) Saline, (B) PDP at 10 mg/kg, (C) PDP at 20 mg/kg, (D) PDP at 40 mg/kg, and (E) the Satellite group (40 mg/kg) (n = 5). Scale bar: 200 μm. No treatment-related pathological abnormalities are observed.

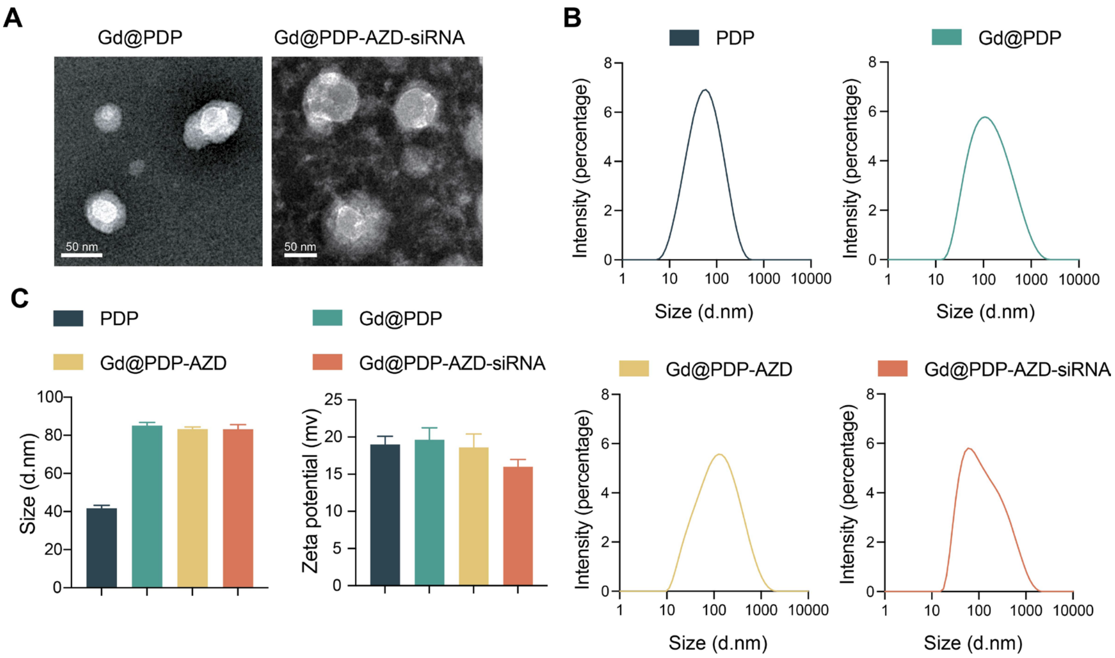

Figure S22. Physicochemical characterization of PDP-derived nanomedicines. (A) TEM images of Gd@PDP and Gd@PDP-AZD-siRNA NPs. Scale bar: 50 nm. (B) Representative DLS size

distribution profiles of PDP, Gd@PDP, Gd@PDP-AZD, and @PDP-AZD-siRNA. (C) The hydrodynamic diameter and zeta potential of PDP, Gd@PDP, Gd@PDP-AZD, and Gd@PDP-AZD-siRNA (n = 3). Mean ± SD.

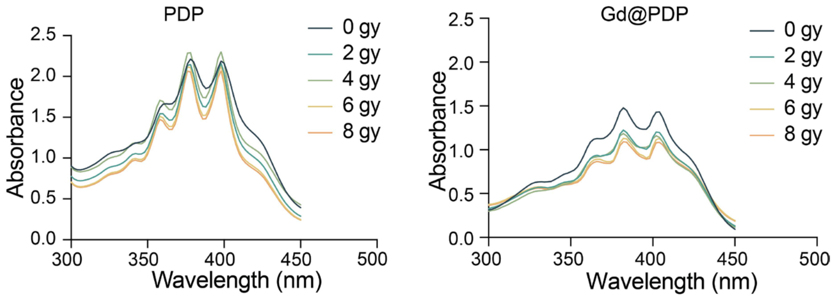

Figure S23. Singlet oxygen generation assessed by ABDA degradation. Gd@PDP shows significantly enhanced radiodynamic activity under X-ray irradiation compared to PDP.

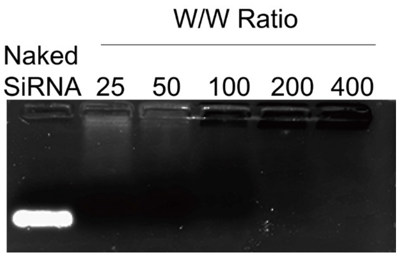

Figure S24. siRNA complexation with Gd@PDP-AZD at various polymer-to-siRNA mass ratios via gel retardation assays.

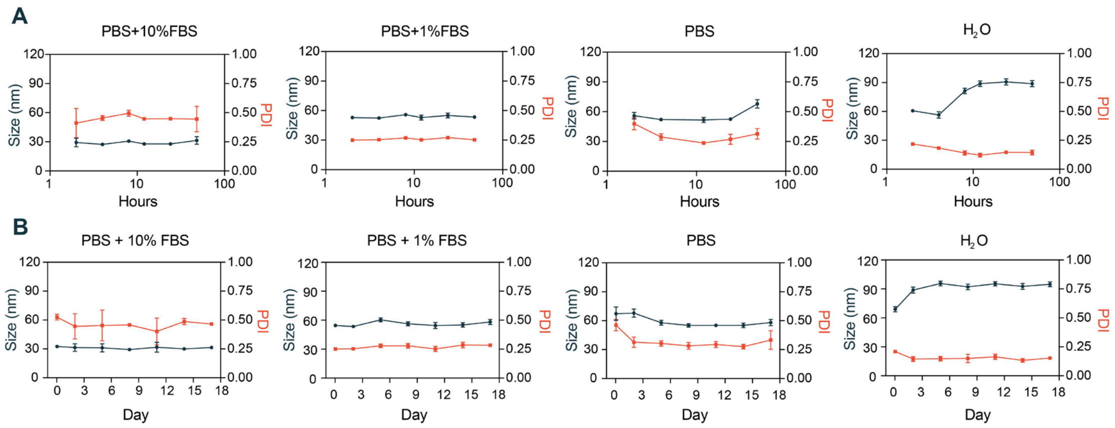

Figure S25. Colloidal and storage stability of Gd@PDP-AZD-siRNA. (A) Hydrodynamic diameter and polydispersity index (PDI) of Gd@PDP-AZD-siRNA in 10% FBS, 1% FBS, PBS, and water at room temperature over 48 h (n = 6). (B) Hydrodynamic diameter and PDI of Gd@PDP-AZD-siRNA during storage at 4 °C over 17 days (n = 6). Mean ± SD.

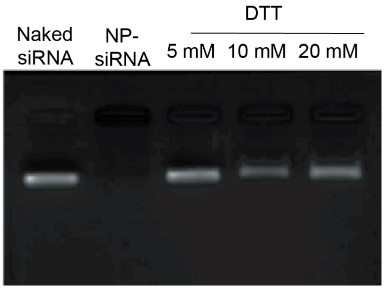

Figure S26. Reductive environment-responsive release of siRNA from Gd@PDP-AZD-siRNA after incubation with DTT via agarose gel electrophoresis.

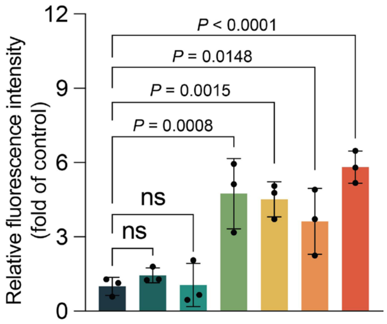

Figure S27. Intracellular ROS levels in A549 cells detected by a DCFH-DA fluorescence probe after treatment with different formulations followed by RT at 4 Gy. ROS levels are presented as relative fluorescence intensity, calculated as the mean ROS fluorescence intensity normalized to the mean nuclear fluorescence intensity and expressed as fold of control (n = 3). Mean ± SD; ns, not significant.

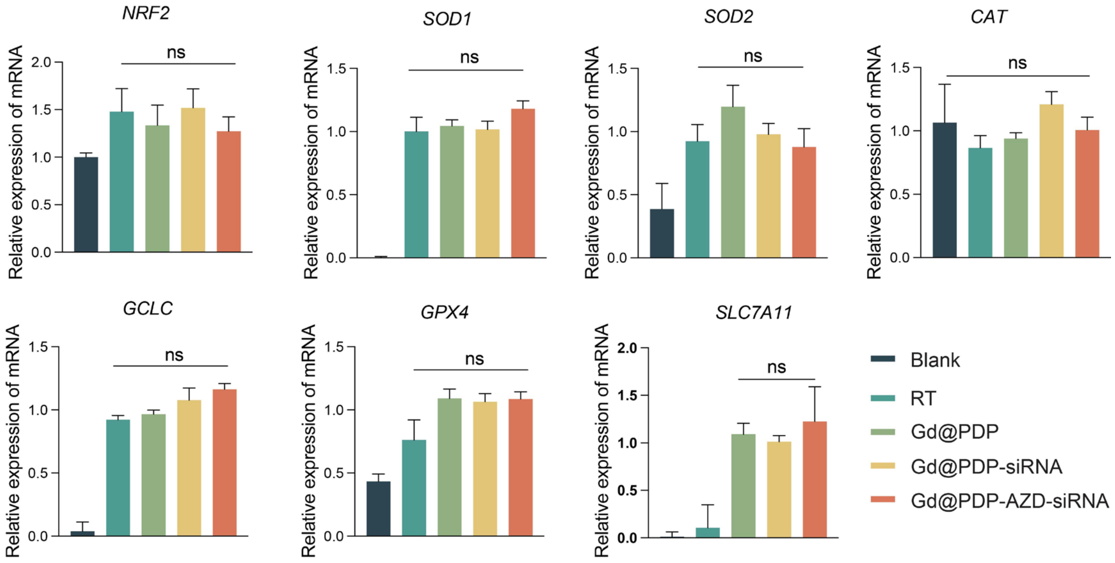

Figure S28. Relative mRNA levels of *NRF2*, *SOD1*, *SOD2*, *CAT*, *GCLC*, *GPX4*, and *SLC7A11* in A549 cells determined by qRT-PCR (n = 3). Mean ± standard error of the mean (SEM); ns, not

significant.

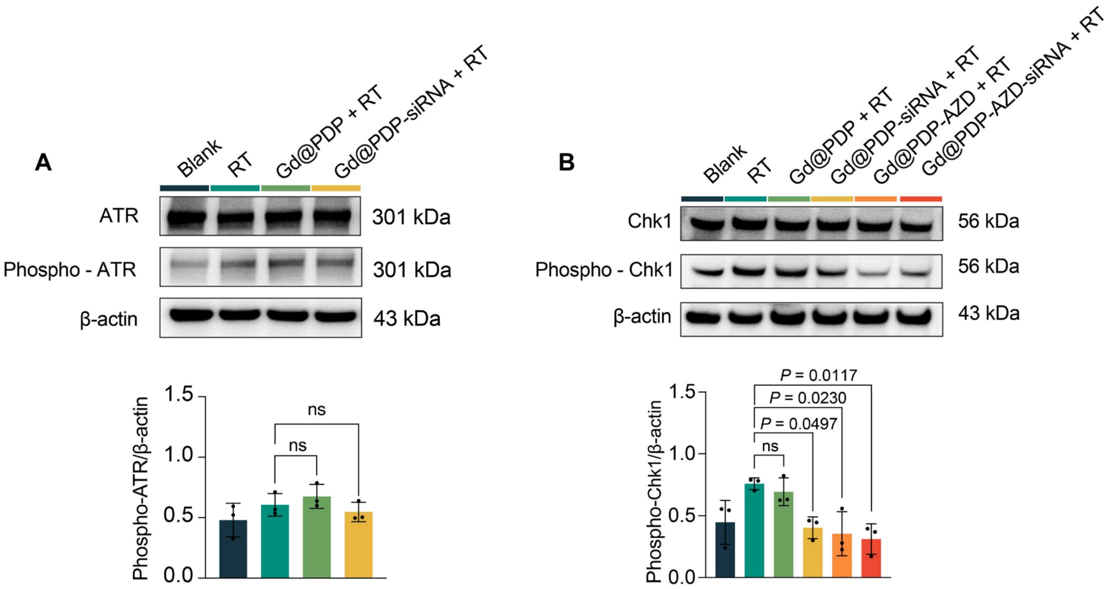

Figure S29. ATR and Chk1 signaling pathway activation. Representative western blots and corresponding quantitative analysis of ATR (ATR and phospho-ATR) and Chk1 (Chk1 and phospho-Chk1) signaling pathway (n = 3). Mean ± SD; ns, not significant.

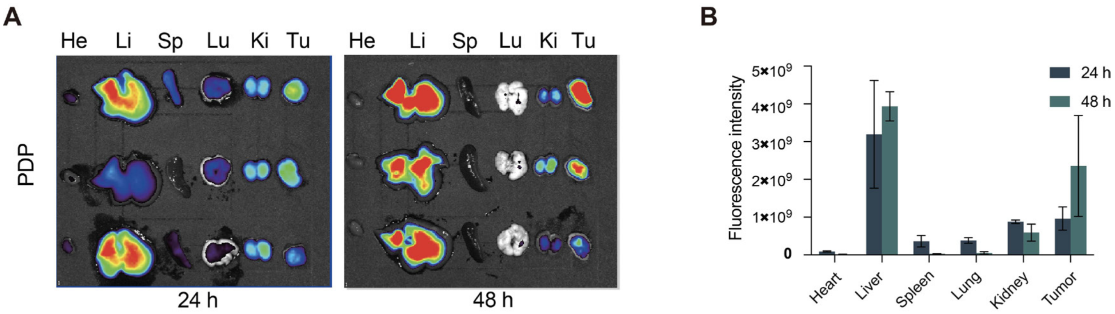

Figure S30. *Ex vivo* tumor targeting of PDP in an LUAD PDX model. (A) *Ex vivo* fluorescence imaging of major organs harvested at 24 h and 48 h after intravenous injection of Gd@PDP. (B) Quantitative analysis of the fluorescence intensity in major organ sections (n = 3). Mean ± SD.

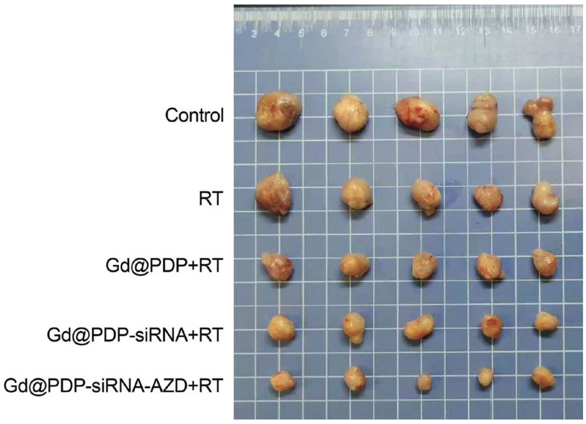

Figure S31. Representative images of tumors at the treatment endpoint (Day 30) (n = 5).

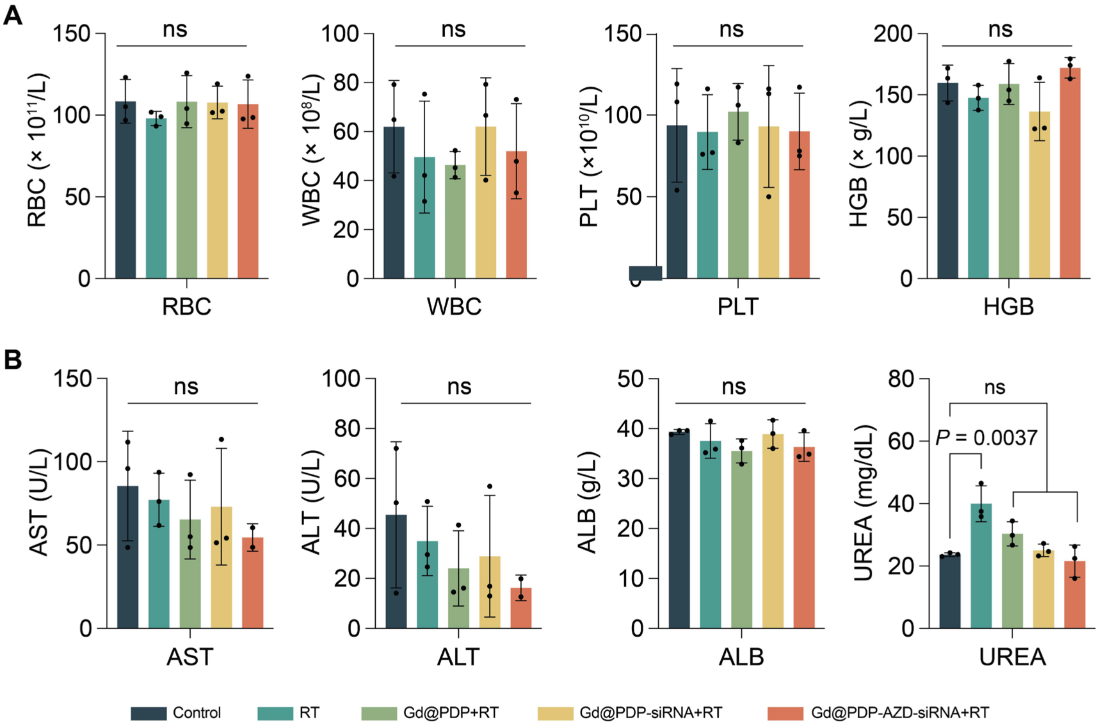

Figure S32. Hematological and serum biochemical analysis of samples from LUAD PDX-bearing mice post-treatment. (A) CBC parameters: red blood cells (RBC), white blood cells (WBC), platelets (PLT), and hemoglobin (HGB) (n = 3). (B) Serum biochemical markers: aspartate aminotransferase (AST), alanine aminotransferase (ALT), albumin (ALB), and urea (UREA) (n = 3). Mean ± SD; ns, not significant.

4. Supplementary tables

Table S1. The hydrodynamic diameter and zeta potential of nanoparticle formulations

Groups	DLS size (nm)	Zeta potential (mV)
PDP	41.73 ± 1.53	19.00 ± 1.10
Gd@PDP	85.06 ± 1.71	19.60 ± 1.63
Gd@PDP-AZD	83.27 ± 1.10	18.60 ± 1.81
Gd@PDP-AZD-siRNA	83.15 ± 2.39	16.01 ± 0.97

Table S2. Body weights of mice during the toxicity study (Mean ± SD, n = 5)

Sex	Dose (mg/kg)	D0 (g)	D8 (g)	D16 (g)	D24 (g)	D28 (g)
Female	0	22.39 ± 0.92	24.12 ± 0.93	24.84 ± 0.66	25.84 ± 0.82	26.19 ± 1.09
	10	22.10 ± 0.93	23.91 ± 1.29	24.26 ± 1.07	24.54 ± 0.83	25.51 ± 0.83
	20	22.01 ± 0.40	23.82 ± 0.41	24.53 ± 0.85	25.91 ± 0.90	26.13 ± 1.11
	40	20.79 ± 0.45	22.91 ± 0.54	23.60 ± 0.44	24.29 ± 0.59	25.04 ± 0.71
	Satellite	20.74 ± 1.48	22.68 ± 1.32	23.35 ± 1.37	24.17 ± 1.29	24.98 ± 1.32
Male	0	21.24 ± 1.17	22.42 ± 1.65	22.86 ± 1.48	23.78 ± 1.77	24.13 ± 1.50
	10	22.18 ± 0.59	22.95 ± 0.55	23.98 ± 0.47	24.19 ± 0.79	24.56 ± 0.80
	20	21.73 ± 0.41	22.59 ± 0.61	22.92 ± 0.41	23.17 ± 0.52	23.43 ± 0.62
	40	20.83 ± 1.38	21.86 ± 1.40	22.05 ± 1.33	22.87 ± 1.67	23.10 ± 1.67
	Satellite	22.17 ± 1.44	22.59 ± 1.56	22.91 ± 1.39	23.20 ± 1.48	23.69 ± 1.58

Table S3 Hematological parameters of mice after the toxicity study (Mean ± SD, n = 5)

Sex	Dose (mg/kg)	HCT (L/L)	HGB (g/L)	LYM (%)	MCH (pg)	MCV (fL)	NEU (%)	PLT (10^9/L)	RBC (10^{12}/L)	WBC (10^9/L)
Female	0	50.56 ± 3.55	168.00 ± 11.92	7.37 ± 2.27	15.78 ± 0.19	46.38 ± 0.97	2.34 ± 0.74	964.40 ±118.12	11.63 ± 0.68	10.25 ± 2.76
	10	49.04 ± 1.18	165.60 ± 3.83	6.93 ± 1.51	15.56 ± 0.30	46.40 ± 0.62	2.28 ± 0.83	953.60 ± 87.27	10.49 ± 0.96	10.90 ± 1.51
	20	49.77 ± 2.29	163.20 ± 5.46	6.22 ± 1.00	15.94 ± 0.22	47.92 ± 0.87	2.36 ± 0.27	934.40 ± 106.20	10.24 ± 0.28	8.90 ± 1.20
	40	52.92 ± 3.37	170.40 ± 2.88	6.53 ± 1.62	16.32 ± 0.30	48.28 ± 0.61	2.20 ± 0.35	989.60 ± 74.45	10.55 ± 1.49	8.89 ± 1.76
	Satellite	51.16 ± 1.78	164.00 ± 9.94	7.43 ± 1.29	15.70 ± 0.32	47.02 ± 0.96	2.18 ± 0.24	958.80 ± 91.73	11.15 ± 1.18	9.24 ± 2.14
Male	0	53.18 ± 3.34	171.80 ± 13.79	7.19 ± 1.75	15.80 ± 0.25	45.70 ± 0.60	2.74 ± 0.63	949.40 ± 152.26	10.91 ± 0.95	10.36 ± 1.49
	10	49.74 ± 4.09	164.60 ± 15.40	6.12 ± 1.57	15.80 ± 0.13	47.42 ± 0.69	2.89 ± 0.43	953.00 ± 150.76	10.57 ± 0.30	9.42 ± 2.74
	20	52.66 ± 3.01	170.60 ± 5.16	6.77 ± 1.13	15.54 ± 0.39	46.66 ± 0.27	2.62 ± 0.28	970.00 ± 80.55	11.28 ± 0.67	9.79 ± 1.29
	40	49.20 ± 1.98	167.40 ± 8.82	6.48 ± 0.89	15.54 ± 0.43	45.76 ± 0.83	3.06 ± 0.50	972.00 ± 117.65	10.54 ± 0.64	10.05 ± 1.00
	Satellite	48.03 ± 3.68	164.75 ± 5.97	7.54 ± 1.57	16.32 ± 0.30	46.68 ± 0.33	2.59 ± 0.17	966.75 ± 110.81	10.88 ± 0.70	10.17 ± 0.49

HCT: Hematocrit; HGB: Hemoglobin; LYM: Lymphocytes; MCH: Mean Corpuscular Hemoglobin; MCV: Mean Corpuscular Volume; Neu: Neutrophils; PLT: Platelets; RBC: Red Blood Cells; WBC: White Blood Cells.

Table S4. Blood biochemical parameters of mice after the toxicity study (Mean ± SD, n = 5)

Sex	Dose (mg/kg)	ALT (U/L)	AST (U/L)	ALB (g/L)	TP (g/L)	GLU (mmol/L)	CREA (mmol/L)	UA (µmol/L)	UREA (mmol/L)
Female	0	13.33 ± 4.20	52.67 ± 5.06	15.33 ± 3.61	2.39 ± 0.36	45.93 ± 7.87	0.13 ± 0.02	0.80 ± 0.16	13.20 ± 1.69
	10	13.73 ± 1.73	51.53 ± 4.73	14.13 ± 1.05	2.45 ± 0.04	46.53 ± 8.73	0.13 ± 0.01	0.93± 0.09	12.30 ± 0.78
	20	13.00 ± 2.59	49.87 ± 7.57	14.47 ± 0.98	2.63 ± 0.16	43.07 ± 2.53	0.13 ± 0.02	0.91 ± 0.12	13.33 ± 1.83
	40	13.03 ± 2.85	50.17 ± 4.46	14.07 ± 0.90	2.49 ± 0.08	44.27 ± 2.92	0.13 ± 0.02	0.89 ± 0.16	12.73 ± 0.57
	Satellite	13.40 ± 2.41	50.20 ± 3.67	14.87 ± 1.40	2.57 ± 0.16	45.67 ± 4.61	0.13 ± 0.01	0.83 ± 0.09	14.80 ± 1.85
Male	0	12.35 ± 2.18	52.47 ± 4.06	15.78 ± 1.21	2.76 ± 0.46	47.23 ± 7.85	0.12 ± 0.01	0.67 ± 0.14	13.60 ± 1.39
	10	12.56 ± 1.72	51.46 ± 4.42	14.30 ± 1.05	2.75 ± 0.02	46.93 ± 6.73	0.13 ± 0.01	0.72 ± 0.08	14.36 ± 0.68
	20	13.10 ± 2.09	48.47 ± 3.58	14.87 ± 0.98	2.61 ± 0.14	48.21 ± 2.33	0.13 ± 0.01	0.81 ± 0.13	13.43 ± 1.86
	40	13.04 ± 2.84	47.68 ± 5.40	14.19 ± 0.89	2.69 ± 0.07	44.07 ± 2.82	0.10 ± 0.01	0.79 ± 0.14	14.73 ± 0.51
	Satellite	12.40 ± 2.30	50.23 ± 3.76	14.69 ± 1.41	2.77 ± 0.17	49.03 ± 4.87	0.13 ± 0.01	0.63 ± 0.09	13.80 ± 1.90

ALT: Alanine Aminotransferase; AST: Aspartate Aminotransferase; ALB: Albumin; TP: Total Protein; GLU: Glucose; CREA: Creatinine; UA: Uric Acid; UREA: Urea.

Table S5. Organ coefficients (%) of mice after the toxicity study (Mean ± SD, n = 5)

Sex	Dose (mg/kg)	Heart	Liver	Spleen	Lung	Kidney
Female	0	1.98 ± 0.29	17.24 ± 0.88	1.26 ± 0.20	2.58 ± 0.39	2.81 ± 0.34
	10	2.00 ± 0.36	16.78 ± 1.21	1.21 ± 0.18	2.57 ± 0.63	2.94 ± 0.15
	20	1.94 ± 0.44	16.00 ± 0.78	1.29 ± 0.16	2.59 ± 0.15	2.92 ± 0.38
	40	2.14 ± 0.28	16.68 ± 1.06	1.31 ± 0.26	2.27 ± 0.42	2.88 ± 0.32
	Satellite	1.96 ± 0.48	17.38 ± 1.23	1.42 ± 0.53	2.54 ± 0.58	2.84 ± 0.47
Male	0	2.05 ± 0.41	18.39 ± 1.14	1.51 ± 0.40	3.04 ± 0.85	3.21 ± 0.38
	10	2.11 ± 0.34	17.38 ± 1.05	1.70 ± 0.16	3.40 ± 0.37	3.01 ± 0.38
	20	2.02 ± 0.49	18.35 ± 1.09	1.65 ± 0.19	3.29 ± 0.46	3.31 ± 0.49
	40	2.35 ± 0.29	18.72 ± 1.35	1.85 ± 0.21	3.31 ± 0.67	3.25 ± 0.42
	Satellite	2.16 ± 0.68	17.53 ± 1.12	1.74 ± 0.23	3.37 ± 0.58	3.47 ± 0.32

References

1. Shen X, Cai H, Wang Y, Xie M, Li Y, Pan D, et al. Metabolic targeting of oxidative phosphorylation enhances chemosensitivity in triple-negative breast cancer via a synergistic nanomedicine. Theranostics. 2025; 15: 7607-26.

2. Huang J, Zhuang C, Chen J, Chen X, Li X, Zhang T, et al. Targeted drug/gene/photodynamic therapy via a stimuli-responsive dendritic-polymer-based nanococktail for treatment of EGFR-TKI-resistant non-small-cell lung cancer. Adv Mater. 2022; 34: e2201516.

3. Cheng X, Cai H, Li X, Zhang Y, Li S, Li Y, et al. Reversing adenosine-mediated immunosuppression in triple-negative breast cancer by synergistic chemo-immunotherapy via stimuli-responsive nanomedicines. EBioMedicine. 2025; 123: 106059.
